# Prenatal stress and child development: A scoping review of research in low- and middle-income countries

**DOI:** 10.1371/journal.pone.0207235

**Published:** 2018-12-28

**Authors:** Giavana Buffa, Salomé Dahan, Isabelle Sinclair, Myriane St-Pierre, Noushin Roofigari, Dima Mutran, Jean-Jacques Rondeau, Kelsey Needham Dancause

**Affiliations:** 1 Ross University School of Medicine, Portsmouth, Dominica; 2 University of Haifa, School of Public Health, Haifa, Israel; 3 UQAM, Département des sciences de l’activité physique, Montréal, Canada; 4 Université Laval, Faculté de pharmacie, Québec City, Canada; 5 École des hautes études commerciales, Département de sciences de la gestion, Montréal, Canada; 6 Université du Québec à Montréal (UQAM), Bibliothèque des Sciences, Montréal, Canada; Temple University, UNITED STATES

## Abstract

**Introduction:**

Past research has shown relationships between stress during pregnancy, and related psychosocial health measures such as anxiety and depressive symptoms, with infant, child, and adult outcomes. However, most research is from high-income countries. We conducted a scoping review to identify research studies on prenatal stress and outcomes of the pregnancy or offspring in low- and middle-income countries (LMICs), and to synthesize the stress measures and outcomes assessed, the findings observed, and directions for future research.

**Methods:**

We searched PubMed, Scopus, and PsycINFO for English-language abstracts published from Jan 1960-Jan 2017. Search terms were related to stress and psychosocial health; pregnancy; infant or child development; and LMICs.

**Results:**

48 articles were identified. Sixty percent of studies were in upper-middle, 25% in lower-middle, and 15% in low income countries. Most studies used questionnaires, either existing or tailor-made, to assess stress. Eight assessed cortisol. Most studies (n = 31) assessed infant outcomes at birth, particularly gestational age or preterm birth (n = 22, 12 showing significant relationships), and birthweight (n = 21, 14 showing significant relationships). Five studies analyzed outcomes later in infancy such as temperament and motor development, all showing significant results; and nine in childhood such as behavioral development, asthma, and physical growth, with eight showing significant results.

**Conclusions:**

Results highlight the importance of prenatal stress on infant and child outcomes in LMICs. Methods used in high-income countries are successfully employed in LMICs, but tailored tools remain necessary. Careful assessment of covariates is needed to foster analyses of interactive effects and pathways. Studies including longer-term follow-up should be prioritized.

## Introduction

Research in the Developmental Origins of Health and Disease has highlighted effects of maternal stress during pregnancy on long-term health outcomes among offspring [1–6]. This reflects in part the effects of maternal stress hormones such as cortisol, which is important in promoting fetal neurodevelopment but which, at high levels, might have adverse effects that vary by infant sex and the timing of exposure during gestation [7–10]. Furthermore, prenatal stress is associated with epigenetic changes in the placenta and fetus that affect both early and later development [11]. Human studies have shown relationships between prenatal stress and birth outcomes, such as low birthweight and preterm birth [1, 10, 12]. Prenatal stress also predicts later cognitive, behavioral, and psychomotor development [10, 13, 14], linguistic development [5], immune function [5], and metabolic outcomes in childhood and persisting throughout life [2].

Health researchers and international organizations such as the World Health Organization often distinguish between high-income countries and low and middle-income countries (LMICs), based on differences in economic indicators that also capture differences in common health risks and burdens [15, 16]. Countries falling below thresholds defined by the World Bank [17] (detailed below) are classified as low, lower-middle, or upper-middle income countries and are together referred to as LMICs. The prenatal stress reviews cited above [1–14] synthesize evidence primarily from industrialized or high-income countries, with little information on developing countries or LMICs. This reflects the fewer number of studies in LMICs [1, 3, 18], difficulty comparing studies because of varied methods [18], and the likelihood of different relationships between prenatal stress and developmental outcomes in LMICs compared to high-income countries, which complicates the synthesis of results [6].

Reviews that do include LMICs highlight the importance of maternal psychosocial health on child developmental outcomes, but also the need for a broader review. For example, one review summarized relationships between perinatal distress and preterm birth in LMICs, with an emphasis on allostatic load [18]. The review provides an excellent overview of allostatic load as a stress indicator, but the focused search yielded only three studies from LMICs. Another review synthesized research on perinatal mental disorders and child outcomes [19], but focused on disorders (anxiety, depression) and did not include search terms for stress. Furthermore, the seven studies from LMICs all focused on postnatal rather than prenatal exposure [19]. Finally, a review of depression during pregnancy [20] identified several studies from LMICs. Whereas some of these also included maternal stress, the aim of the review was to synthesize research specifically on depression, and thus a scoping review of prenatal stress remains necessary.

Although there is wide variability among countries, developing countries and LMICs share many similar challenges in healthcare funding [15] and provision of services [16], as well as maternal and infant health burdens such as low birthweight and prematurity. For example, prevalence of low birthweight is 18.6% in least developed countries and 16.5% in less developed, compared to only 7.0% in more developed countries [21]. Given these persistent disparities in outcomes associated with prenatal stress, there is a growing interest in incorporating prenatal stress into global health research [22] and better integrating maternal psychosocial health into prenatal care in LMICs [23]. A review that synthesizes the literature more broadly could highlight existing datasets, commonly assessed stress and outcome measures, research challenges, and gaps in knowledge to guide future research. Thus, our objective was to review research in LMICs on stress and related maternal psychosocial health measures during pregnancy, and child developmental outcomes, in order to summarize the stress measures and outcomes assessed, the general relationships observed, and priorities for future research.

## Materials and methods

The search strategy targeted studies assessing relationships between stress and related psychosocial health outcomes during pregnancy, and any child developmental outcome. S1 Table shows the full search strategy. We searched PubMed, Scopus, and PsycINFO for English-language abstracts published between Jan 1, 1960, and Jan 31, 2017. Search terms included those related to stress, distress, stressful life events, and psychosocial health; pregnancy; infant or child development; and developing countries or LMICs, including countries classified by the World Bank [17] in 2017 as low income (Gross National Income per capita<$1,025 USD), lower-middle income ($1,026–$4,035), or upper-middle income ($4,036–$12,475). The full list of country classifications is available in S1 Table. Reviewed articles were limited to English. Articles were excluded if they did not include a stress or psychosocial health measure, did not assess outcomes of the pregnancy or offspring, were not in LMICs, presented animal models, or focused on in vitro fertilization.

S1 Appendix presents the PRISMA checklist for the current study.

## Results

**Fig 1:**
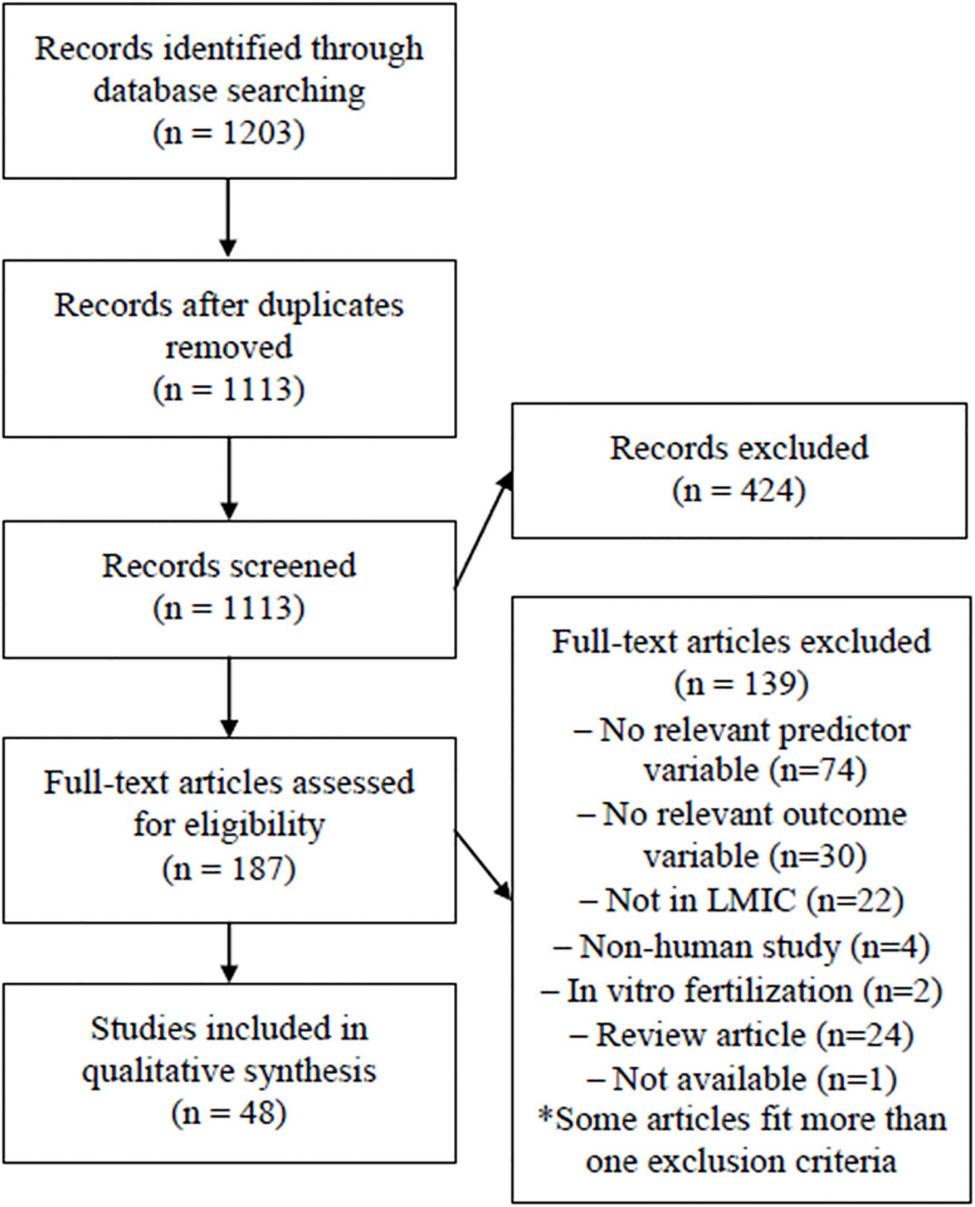
Flow diagram of search strategy and article selection.

The search strategy yielded 1113 abstracts after removal of duplicates (Fig 1). Initial screening of descriptors resulted in exclusion of 424 abstracts. Review for keywords in titles and abstracts yielded 187 articles for complete review (Fig 1). One article deemed likely pertinent [24] was not available despite attempts to contact the journal and authors. Thus, 186 articles were reviewed in detail by at least one author and discussed by at least two to establish consensus of pertinence. Articles were excluded if they did not include a stress or psychosocial health measure (n = 74). This was the case for many studies of stress following war (n = 21) and natural disasters (n = 10) in which, without a measure of psychosocial health, it is not possible to isolate the effects of stress from those of other factors such as infectious diseases or undernutrition that might be more prevalent during such events and that might also affect child development. Other studies were excluded because they did not assess outcomes of the pregnancy or offspring (n = 30); they were not in LMICs (n = 22); they presented animal models (n = 4); or they focused on in vitro fertilization (n = 2), which was deemed not pertinent to the current review. Some articles fit more than one exclusion category. Finally, 24 reviews were excluded.

The review process highlighted the use of questionnaires to assess common mental disorders, such as anxiety or depression, as a construct of stress and psychosocial health more generally. This reflects the difficulty of distinguishing between constructs that share many similar underlying mechanisms of influence, which might be particularly challenging in the context of LMICs lacking detailed and validated tools to evaluate stress specifically. We thus chose to retain articles that used measures of common mental disorders as a generalized conceptualization of stress or distress. However, we group results by the measure employed, for distinction.

Forty-eight articles were retained, and were read and summarized by at least two authors. Table 1 summarizes the country in which each study was conducted, measures of stress examined, outcomes, and results. S2 Table includes these information as well as detailed descriptions of the sample, data collection, analyses, and discussion points highlighting strengths and limitations. S2 Appendix presents methodological quality assessment for individual studies, based on the Quality Assessment Tool for Quantitative Studies developed by the Effective Public Health Practice Project.

**Table 1:** Summary of studies of prenatal stress and pregnancy and child health outcomes in LMICs.

Article	Country	Sample	Stress measure	Outcome measures	Results
Abeysena et al. 2010 (35)	Sri Lanka (South Asia, LM income)	737 pregnant women	Modified Life Events Inv. (MLEI), General Health Quest. (GHQ)	PTB	GHQ and MLEI scores were not significant predictors of PTB. Multivariate analyses indicated a trend for exposure to MLEIs during the 2^nd^T and PTB (p = 0.09, OR 1.80).
Abramson et al. 1961 (62)	South Africa (Sub-Saharan Africa, UM income)	101 pregnant women	Interview: Feelings about pregnancy, life events, family relationships	Neuromotor development 3, 32, and 93 days after delivery	Excluding babies with BW>8.0 pounds, 42.5% in the high-stress group had motor scores <3, compared to 15.4% in the low-stress group. Results were similar at the 2^nd^ but not 3^rd^ evaluation.
Arffin et al. 2012 (56)	Malaysia (East Asia & Pacific, UM income)	33 pregnant women with history of miscarriage and PTB (n = 20) or controls (n = 13)	Salivary cortisol	History of miscarriages, PTB	Mean salivary cortisol levels did not differ between the test (1.016±SEM 0.182 lg/ml) and control (0.978±0.298 lg/ml) groups (p = 0.392).
Arteaga-Guerra et al. 2010 (29)	Colombia (Latin Am. & Caribbean, UM income)	46 perinatal women	Perceived Stress Scale	PTB, LBW, PTB & LBW combined (PLBW)	Perceived stress was not associated with PTB or LBW. The combination of periodontitis but no elevated stress predicted PLBW (OR = 10.3, CI = 1.1, 93.2, p = 0.01).
Baig et al. 2013 (43)	Pakistan (South Asia, LM income)	600 women with preterm (n = 300) or term (n = 300) births	Edinburgh Postnatal Depression Scale	PTB	Prevalence of emotional stress was 69% among cases and 55% among controls (p<0.01). Risk factors for PTB included maternal weight <50 kg, periodontal diseases, low haemoglobin, history of PTB, and poor nutritional status.
Barrios et al. 2014 (31)	Peru (Latin Am. & Caribbean, UM income)	959 women with preterm (n = 479) or term (n = 480) births	Interview: Life events	PTB	PTB was associated with severe life events including death of a relative (OR = 2.10, CI = 1.38, 3.20), divorce/separation (OR = 2.09, CI = 1.10, 4.00), financial troubles (OR = 2.70, CI = 1.85, 3.94), and fights with partner (OR = 2.40, CI = 1.78, 3.17). Risk increased with # of life events.
Başgül et al. 2011 (69)	Turkey (Europe & Central Asia, UM income)	309 children age 3–5 yrs: 204 controls, 105 with psychiatric complaints	Investigator-prepared form: Stressors experienced by the mother	Early Childhood Inventory-4 Parent Form, DSM-IV interviews	No differences in stress or other predictor variables were found between children with psychiatric conditions and those without.
Bhat et al. 2015 (59)	India (South Asia, LM income)	100 pregnant women	General Health Quest. (GHQ-28)	Early Infancy Temperament Questionnaire at 1–4 months of age	GHQ scores were not correlated with temperament. Mean adaptability and approach scores were higher among infants of mothers with GHQ<7 (3.11 and 2.70) compared to those with GHQ≥7 (2.67, 2.06) (p = 0.03, p = 0.05). Other temperament dimensions did not differ between groups.
Bindt et al. 2013 (40)	Ghana & Cote d’Ivoire (Sub-Saharan Africa, LM income)	719 pregnant women	Patient Health Quest., Generalized Anxiety Disorder Scale	BW, LBW, head circumference, GA, PTB, Apgar score	Anxiety and depression scores were weakly correlated with Apgar scores (r = -0.106 and -0.102, respectively) but not with BW or GA. Depression and anxiety were not predictive of BW or PTB.
Brittain et al. 2015 (41)	South Africa (Sub-Saharan Africa, UM income)	726 pregnant women	Beck Depression Inv., World Mental Health Life Events Quest.	BW, HC, WAZ, HCAZ, SGA	No associations were observed between antenatal depression and PTB. Antenatal depression predicted smaller WAZ (OR = 0.2, CI = 0.02, 0.4) and HCAZ (OR = 0.3, CI = 0.1, 0.6). Relationships between depression and WAZ did not persist when controlling for stressful life events.
Cerón-Mireles et al. 1996 (44)	Mexico (Latin Am. & Caribbean, UM income)	2623 perinatal women	Karasek’s Job Content Quest.	GA, PTB, BW, SGA	Conflicts at work predicted SGA only among women who delivered at the public assistance hospital, which typically sees the poorest women (OR = 4.93, CI = 2.09, 11.66).
Chen et al. 2000 (53)	China (East Asia & Pacific, UM income)	792 pregnant women exposed to benzene (n = 354) or not exposed (n = 438)	Perceived work stress: single questionnaire item	BW	Adjusted mean BW was 3445g among the group with neither work stress nor benzene exposure, 3426g with work stress alone, 3430g with benzene exposure alone, and 3262g with both exposures.
Christian et al. 2016 (36)	Nepal (South Asia, low income)	737 pregnant women assigned to one of 5 supplement groups	Serum cortisol	GA, LBW, PTB	Cortisol predicted PTB (OR = 1.04, CI = 1.00, 1.08), but not BW (*β* = 4.2, p = 0.17) or LBW (OR = 0.98, CI = 0.95, 1.01).
Fan et al. 2016 (70)	China (East Asia & Pacific, UM income)	216 pregnant women	Hamilton Anxiety Scale, Hamilton Rating Scale for Depression	Resting blood pressure (BP) and heart rate (HR) at age 7–9 years	Maternal anxiety predicted all child HR and BP measurements in multivariate analyses. Maternal depression predicted children’s resting HR, stress systolic BP, and recovery systolic and diastolic BP.
Frith et al. 2015 (37)	Bangladesh (South Asia, LM income)	1041 women assigned to “early” or “usual start” supplementation during pregnancy	Salivary cortisol	BW, birth length (BL), head circumference (HC), GA	Male (but not female) infants of mothers with higher cortisol had smaller BW, HC, and GA. In general linear models, relationships between cortisol and BW and HC differed by supplementation group. In the “usual start” group, greater cortisol predicted smaller values. In the “early start” group, cortisol did not predict outcomes.
Hanlon et al. 2009 (48)	Ethiopia (Sub-Saharan Africa, low income)	1065 pregnant women, 521 singleton infants	Self-Reporting Quest. (SRQ-20) of common mental disorders (CMDs), List of Threatening Experiences Quest.	BW, stillbirth, neonatal mortality, prolonged labor, time to initiation of breast-feeding	There were no associations between BW, stillbirth, or neonatal death and CMD symptoms, stressful life events, or worry in pregnancy. Prolonged labor was associated with CMD symptoms (Low, RR = 1.4, CI = 1.0, 1.9; High, RR = 1.6, CI = 1.0, 2.6) and worry about the delivery (RR = 1.5, CI = 1.0, 2.1). Analyses did not support a linear effect of SRQ score.
Isaksson et al. 2015 (64)	Nicaragua (Latin Am. & Caribbean, LM income)	147 pregnant women, 70 children	Salivary cortisol, Self- Reporting Quest. (SRQ-20)	Child’s psychiatric symptoms at age 9 years (CBCL), child’s salivary cortisol	Morning cortisol during pregnancy was associated with total CBCL scores (r = 0.31, p = 0.009), and with internalizing (r = 0.28, p = 0.020) and externalizing symptoms (r = 0.35, p = 0.003). Cortisol during pregnancy did not correlate with children’s cortisol at age 9. SRQ was not associated with CBCL scores or children’s cortisol.
Karamoozian & Askarizaden 2015 (54)	Iran (Middle East & North Africa, UM income)	29 pregnant women with anxiety or depression assigned to stress management intervention (n = 14) or controls (n = 15)	Edinburgh Postnatal Depression Scale, Pregnancy-Related Anxiety Quest.	Apgar scores	Mean one-minute Apgar scores were higher in the experimental (8.93) than control (8.07) group (p = 0.01). Similar results were observed at 5 minutes (9.71 and 9.27, respectively; p = 0.05).
Kertes et al. 2016 (57)	Democratic Republic of Congo (Sub-Saharan Africa, low income)	24 mother-newborn dyads	Semi-structured ethnographic interviews: Stressful events during pregnancy	Methylation of CRH, CRHBP, NR3C1, and FKBP5, from maternal venous blood, placenta, and umbilical cord blood	18 CpG sites were associated with either chronic stress (n = 11), war trauma (n = 14), or both (n = 6). Correcting for multiple testing, 8 CpG sites remained, all associated with war trauma. Methylation levels at four CpG sites, situated at transcription factor binding sites in NR3C1 and CRH, collectively explained 55% of the variance in BW.
Koen et al. 2016 (45)	South Africa (Sub-Saharan Africa, UM income)	544 pregnant women	Childhood Trauma Quest., Mini Int. Neuropsychiatric Interview (MINI) to assess PTSD	WAZ, HCAZ, SGA, PTB	PTSD did not predict WAZ, HCAZ, SGA, or PTB. Lifetime trauma exposure (MINI) predicted a 0.3 unit reduction in HCAZ (p = 0.026). Trauma did not predict most outcomes.
Meghea et al. 2014 (25)	Romania (Europe & Central Asia, UM income)	474 pregnant women	Perceived Stress Scale (PSS-4)	BW, GA, SGA, PTB	High stress predicted a 113g reduction in BW (CI = -213, -11), and predicted PTB (OR = 2.81, CI = 1.17, 6.76).
Mirabzadeh et al. 2013 (34)	Iran (Middle East & North Africa, UM income)	550 pregnant women	Depression Anxiety Stress Scales (DASS-21), Stressful Life Events Quest.	PTB	GA was negatively correlated with depression, anxiety, stress, and DASS scores. Path analysis showed direct relationships among DASS and GA (β = -0.18).
Mulligan et al. 2012 (58)	Democratic Republic of Congo (Sub-Saharan Africa, low income)	25 mother-newborn dyads	Ethnographic interviews: deprivation, “mundane stressors”, war stressors; Peritraumatic Distress Inv.	Methylation of NR3C1 promoter, from maternal venous and umbilical cord blood; BW	BW was correlated with maternal deprivation (r = -0.484), mundane stress (r = -0.521), and war stress (r = -0.620). War stress was correlated with cord blood methylation levels of the NR3C1 gene (r = 0.586) and BW (r = -0.449). Relationships between war stress and maternal methylation, and between maternal methylation and BW, were not significant.
Nasiri et al. 2010 (38)	Iran (Middle East & North Africa, UM income)	600 pregnant women	Spielberger State-Trait Anxiety Inv.	PTB, LBW	State anxiety score ≥45 was associated with PTB (RR = 3.1, CI = 2.1, 4.7) and LBW (RR = 2.6, CI = 1.6, 4.2).
Nasreen et al. 2010 (49)	Bangladesh (South Asia, LM income)	583 pregnant and postpartum women	Edinburgh Postnatal Depressive Scale, State Trait Anxiety Inv.	LBW	Depressive symptoms predicted LBW (OR = 2.24, CI = 1.37, 3.68). Results were similar for anxiety (OR = 2.08, CI = 1.30, 3.25).
Nepomnaschy et al. 2006 (55)	Guatemala (Latin Am. & Caribbean, LM income)	61 women of reproductive age	Urinary cortisol	Pregnancy: successful or unsuccessful	Mean cortisol levels were higher in unsuccessful than in successful pregnancies. Increased cortisol predicted pregnancy loss (OR = 2.7, CI = 1.2, 6.2). Unsuccessful pregnancies presented a larger proportion of cortisol peaks than successful ones.
Pires et al. 2013 (65)	Brazil (Latin Am. & Caribbean, UM income)	370 children ages 6–13 years	Questionnaire: Mothers were asked “whether the pregnancy was a peaceful time or marked by discord and arguments”	CBCL Teacher Report Form of ADHD symptoms	Discord during pregnancy predicted mother-reported ADHD (OR = 4.54, CI = 2.16, 9.57), but not teacher reported ADHD. Other predictors for mother-reported ADHD included family functioning, social support, and life events in the past year.
Qiao et al. 2012 (42)	China (East Asia & Pacific, UM income)	463 pregnant women	Hospital Anxiety and Depression Scale	Obstetric outcomes	Prevalence of prolonged pregnancy was higher in the Symptom (n = 3, 8.3%) than in the Symptomless group (n = 5, 1.6%) (RR = 4.08, CI = 1.25, 13.33). Other obstetric and neonatal outcomes did not differ among groups.
Qu et al. 2016 (46)	China (East Asia & Pacific, UM income)	2189 pregnant women with PTB (n = 130) or term births (n = 2059)	Revised Pregnancy Stress Rating Scale	PTB	High pregnancy specific stress predicted PTB (RR = 2.92, CI = 1.12, 7.58). Low and medium levels of pregnancy-specific stress were not significant predictors of PTB.
Ramchandani et al. 2010 (67)	South Africa (Sub-Saharan Africa, UM income)	953 pregnant women	Interviews: Marital, family, economic, and societal stress and violence	Richman Behaviour Screening Questionnaire at ages 2 and 4	Child behavior scores at age 4 were higher among children in the high (5.4) compared to low (4.4) prenatal stress group. Prenatal stress predicted behavioral problems at age 4 (OR = 2.66, CI = 1.28, 5.54).
Rondó et al. 2003 (27)	Brazil (Latin Am. & Caribbean, UM income)	865 pregnant women	Perceived Stress Scale (PSS), General Health Quest. (GHQ), State Trait Anxiety Inv. (STAI)	LBW, PTB, intra-uterine growth restriction (IUGR)	GHQ >3 in the 2^nd^ interview predicted LBW (RR = 1.97, CI = 1.12, 3.47), and GHQ >3 in the 3^rd^ interview predicted PTB (RR = 2.32, CI = 1.18, 4.60). Maternal psychosocial health did not predict IUGR. STAI and PSS scores did not predict any outcomes.
Rondó et al. 2013 (71)	Brazil (Latin Am. & Caribbean, UM income)	409 pregnant women and their children ages 5–8 years	Perceived Stress Scale (PSS), General Health Quest. (GHQ), State Trait Anxiety Inv.	BMI Z-scores at ages 5–8 years	PSS scores 5–8 years postpartum (β = -0.04) and 2^nd^T GHQ scores (β = -0.09) predicted children’s BMIZ scores. Thus, greater maternal stress and distress both during pregnancy and postpartum predicted lower child BMIZ scores.
Rosa et al. 2016 (72)	Mexico (Latin Am. & Caribbean, UM income)	417 pregnant women	Crisis in Family Systems Revised survey of negative life events (NLEs)	Asthma, wheeze	NLEs predicted risk of ever wheeze (RR = 1.08, CI = 1.00, 1.16) and wheeze in the past 12 months (RR = 1.12, CI = 1.00, 1.26).
Ross et al. 2011 (63)	Ethiopia (Sub-Saharan Africa, low income)	954 perinatal women	Self-Reporting Quest. of common mental disorders (CMDs)	Infant illness episodes since birth	Persistent CMD symptoms predicted infant diarrhoea (RR = 2.15, CI = 1.39, 3.34). Univariate analyses showed relationships between persistent CMD and acute respiratory infection (crude RR = 2.24, CI = 1.52, 3.30) and fever (crude RR = 1.61, CI = 1.10, 2.35), but results did not persist in multivariate analyses.
Rothberg et al. 1991 (47)	South Africa (Sub-Saharan Africa, UM income)	1197 perinatal women, delivering at Johannesburg or Baragwanath Hospital	Social Readjustment Rating Scale (SRRS) of life events	BW	Among the Johannesburg group, greater SRRS predicted BW. SRRS scores did not predict BW in the Baragwanath group. Maternal perception of stress and psychosocial support did not predict BW.
Ruwanpathirana & Fernando 2014 (50)	Sri Lanka (South Asia, LM income)	835 pregnant women and their infants: 167 SGA, 668 controls	General Health Quest. (GHQ-30)	SGA	High 2^nd^T stress levels predicted SGA in univariate (OR = 2.17, CI = 1.43, 3.30) and multivariate (OR = 1.92, CI = 1.17, 3.14) analyses.
Sanchez et al. 2013 (39)	Peru (Latin Am. & Caribbean, UM income)	959 pregnant women: 479 with PTB, 480 with term births	Patient Health Quest. (PHQ-9), Depression Anxiety Stress Scales (DASS-21)	PTB	Predictors of PTB included PHQ-9 Depression scores (OR for moderate-severe group = 3.67, CI = 2.09, 6.46), and DASS-21 Depression (OR = 2.90, CI = 1.66, 5.04), Anxiety (OR = 2.76, CI = 1.83, 4.16), and Stress (OR = 11.07, CI = 5.64, 21.71) scores.
Sanguanklin et al. 2014 (52)	Thailand (East Asia & Pacific, UM income)	175 pregnant women	Center for Epidem. Studies Depression Scale	BW, LBW, PTB	Depression had no main effects on BW.
Santos et al. 2014 (68)	Brazil (Latin Am. & Caribbean, UM income)	4231 perinatal women (2004 Pelotas Birth Cohort)	Perinatal interview: “During pregnancy, did you feel depressed or have any nervous condition?”	Dev. & Well-Being Assessment questionnaire (psychiatric disorders) at age 6	Mood symptoms during pregnancy predicted psychiatric disorders among children (OR = 1.97, CI = 1.60, 2.41). Results were similar for mood symptoms at 3 months postpartum (OR = 2.29 CI = 1.86, 2.81).
Sasaluxnanon & Kaewpornsawan 2005 (66)	Thailand (East Asia & Pacific, UM income)	241 children age 6–12: 122 with ADHD, 119 controls	Questionnaire: “Emotional distress” during pregnancy, reported as “yes” or “no”.	Attention Deficit Hyperactivity Disorder (ADHD)	Emotional distress predicted ADHD in univariate (OR = 4.49, CI = 2.37, 8.45) and multivariate analyses (OR = 2.99, CI = 1.43, 5.40).
Shaikh et al. 2011 (30)	Pakistan (South Asia, LM income)	125 pregnant women	A-Z stress scale, Center for Epidem. Studies Depression scale, serum cortisol	PTB	There were no relationships between PTB and cortisol (OR per 10-point increase in cortisol = 0.78, p = 0.507), high stress (OR = 0.60, p = 0.519) or depression (OR = 1.4, p = 0.697)
Stewart et al. 2015 (26)	Malawi (Sub-Saharan Africa, low income)	1391 pregnant women enrolled into one of 3 supplement groups	Perceived Stress Scale (PSS-10), salivary cortisol	BW, BL, HC, arm circumference, WAZ, LAZ, HCZ	Greater cortisol at enrolment and 36 wks predicted shorter GA. Greater cortisol at 28 and 36 wks predicted lower BW. Cortisol was not associated with WAZ, LAZ, or HCZ at any time. PSS scores at 28 and 36 wks predicted shorter LAZ. PSS did not predict other outcomes.
Tran et al. 2014 (60)	Vietnam (East Asia & Pacific, LM income)	378 pregnant women	Edinburgh Postnatal Depression Scale to assess common mental disorders (CMDs)	Bayley Scales of Infant and Toddler Development Social-Emotional Questionnaire	Antenatal CMDs predicted Bayley social-emotional scores (β = -3.30, CI = -6.32, -2.89). Path analyses revealed no direct effects, but a significant indirect effect (estimate = -0.61, CI = -1.17, -0.04): antenatal CMDs predicted postnatal CMDs, which then predicted social-emotional scores.
Valladares et al. 2009 (28)	Nicaragua (Latin Am. & Caribbean, LM income)	147 pregnant women	Salivary cortisol, intimate partner violence, emotional distress, social resources	LBW, PTB, SGA	Increased cortisol predicted LBW and SGA, but not PTB. Women who reported violence had greater prevalence of LBW and SGA, but not PTB. Path analyses suggested that violence predicted LBW through: 1) increased cortisol; 2) increased cortisol and subsequent lower GA; and 3) direct abdominal trauma, and subsequent lower GA.
Wado et al. 2014 (51)	Ethiopia (Sub-Saharan Africa, low income)	537 pregnant women	Edinburgh Postnatal Depression Scale	LBW	Incidence of LBW was 17.9% and was higher among women with depressive symptoms (26.2%) compared to non-symptomatic women (15.8%) (p = 0.01). Antenatal depression predicted LBW (OR = 1.87, CI = 1.09, 3.21).
Zhang et al. 2012 (32)	China (East Asia & Pacific, UM income)	2782 pregnant women: 1391 with PTB, 1391 controls	Questionnaire: Life events (details not provided)	PTB	Stressful life events predicted PTB (OR = 5.54, CI = 2.34, 13.23).
Zhu et al. 2014 (61)	China (East Asia & Pacific, UM income)	152 pregnant women: 38 exposed to severe life events, 114 controls	19-item Prenatal Life Events Checklist	Bayley Mental (MDI) & Psychomotor Dev. Index (PDI), Toddler Temperament Scale (TTS)	Exposure to life events in the 1^st^T predicted lower MDI scores (adjusted mean = 103.11 among cases, 110.09 among controls). Life events did not predict PDI scores. 1^st^T life events predicted higher TTS regularity (adjusted means = 2.77 vs. 2.52) and persistence and attention span scores (adjusted means = 3.61 vs. 3.35).
Zhu et al. 2010 (33)	China (East Asia & Pacific, UM income)	1800 pregnant women	19-item Prenatal Life Events Checklist	PTB, BW	Life events predicted PTB in the 1^st^T (RR = 2.60, CI = 1.29, 5.22) and 2^nd^T (RR = 2.86, CI = 1.32, 6.22), but not the 3^rd^T. Life events during the 1^st^T, but not the 2^nd^ and 3^rd^, predicted BW (RR = -122.97, CI = –166.64, -79.29).

Given the variability in samples, stress measures, and outcomes, calculation of effect sizes for direct comparison among studies was not possible. Measures provided by the authors such as group means, odds ratios, risk ratios, or percentage of variability explained are included for contextualization.

### Countries represented

Twenty-four countries were represented, including four in East Asia and Pacific region (China = 7 studies, Malaysia = 1, Thailand = 2, Vietnam = 1), two in Europe and Central Asia (Romania = 1, Turkey = 1), six in Latin America and the Caribbean (Brazil = 4, Columbia = 1, Guatemala = 1, Mexico = 2, Nicaragua = 2, Peru = 2), one in Middle East and North Africa (Iran = 3), five in South Asia (Bangladesh = 2, India = 1, Nepal = 1, Pakistan = 2, Sri Lanka = 2), and six in Sub-Saharan Africa (Côte d’Ivoire = 1, Democratic Republic of Congo = 2, Ethiopia = 3, Ghana = 1, Malawi = 1, South Africa = 5). One study included samples from two countries. Sixty percent of studies were in upper-middle, 25% in lower-middle, and 15% in low income countries.

### Stress measures analyzed

The stress measures varied widely. Most authors used questionnaires, either tailor-made or existing, such as the Perceived Stress Scale, the Modified Life Events Inventory, or the Depression Anxiety Stress Scales. Others used questionnaires of common mental disorders or affective state such as the Patient Health Questionnaire, Self-Reporting Questionnaire, General Health Questionnaire, Edinburgh Postnatal Depression Scale, Beck Depression Inventory, Center for Epidemiological Studies Depression Scale, and State-Trait Anxiety Inventory. These were often presented as a construct of stress or psychological distress, and were thus retained in the review. Stress measures are categorized below into perceived stress (n = 11), life events (n = 13), depression, anxiety, and common mental disorders (n = 23), occupational stress (n = 2), and trauma (n = 3). Eight others analyzed cortisol, a stress hormone. Many included multiple measures.

### Outcomes analyzed

Most studies (n = 31) assessed infant outcomes at birth, particularly gestational age or preterm birth (n = 22), and birth weight, low birthweight, or small for gestational age (n = 21). Six analyzed other outcomes of the pregnancy or at birth, such as Apgar scores, miscarriage, or methylation of maternal and fetal tissues. Finally, 14 studies analyzed outcomes later in infancy (n = 5) and childhood (n = 9), such as behavioral development, illnesses, and growth measures.

#### Gestational age and preterm birth

Twenty-two studies (46%) analyzed gestational age or preterm birth (PTB). Twelve of these (55%) showed significant associations with at least one stress measure.

Perceived stress: One of six studies showed significant relationships. Studies of 474 Romanian women [25] analyzed Perceived Stress Scale scores within the context of a smoking cessation program. In multivariate regressions, high stress predicted PTB (OR = 2.81, CI = 1.17–6.76). Three others showed no effect of perceived stress, but highlighted other important relationships. For example, studies of 1391 women in Malawi [26] showed relationships between cortisol and PTB (discussed below), but no relationships between Perceived Stress Scale scores and cortisol or gestational age. Similarly, studies of 865 Brazilian women [27] showed that Perceived Stress Scale scores did not predict PTB, but highlighted relationships between General Health Questionnaire scores and PTB (discussed below). Finally, studies of 147 women from Nicaragua [28] showed that perceived stress, based on tailored interviews, predicted increased cortisol, but this was not related to PTB.

Two other studies that did not show significant results suffered from small sample sizes. Studies of 46 women from Columbia [29] analyzed combinations of periodontitis and Perceived Stress Scale scores 48 hours postpartum, and found that Perceived Stress Scale scores did not predict PTB, but the small sample limits analyses. Similarly, case-control studies of 125 women from Pakistan [30] showed no relationships between high stress defined by A-Z Stress Scale scores, and PTB (OR = 0.60, p = 0.519), but the small number of preterm births (n = 15) limits analyses.

Life events: Four of five studies showed significant relationships. Case-control studies in Peru [31] showed that life events such as death of a relative, marital discord, and financial troubles predicted PTB, with ORs ranging from 2.09–2.70. Similar case-control studies in China [32] showed that stressful life events such as hospitalization, death of a relative, and family conflict predicted PTB (OR = 5.54). A second study in China [33] showed that stressful life events predicted PTB in the 1^st^T (RR = 2.60) and 2^nd^T (RR = 2.86), but not the 3^rd^T. Finally, studies of 550 women in Iran [34] showed that whereas anxiety and depression had the greatest direct effects on PTB, stressful life events predicted anxiety and depression and were thus indirectly related to PTB. A fifth study from Sri Lanka [35] failed to show significant relationships, likely because it was underpowered. Results nevertheless showed a trend between 2^nd^T life events and PTB.

Cortisol: Three of five studies showed significant relationships. Studies in Nepal [36] demonstrated that 3^rd^T cortisol predicted PTB (OR = 1.04). Similarly, studies in Bangladesh [37] showed that high 3^rd^T cortisol predicted shorter gestational age among male but not female infants. Finally, studies of 1391 women in the International Lipid-based Nutrient Supplement study in Malawi [26] showed that greater cortisol at <20 and 36 weeks pregnancy predicted smaller gestational age. Two others showed no significant relationships. Studies of 125 women in Pakistan [30] showed no differences in blood cortisol levels from 28–30 weeks pregnancy between women who delivered preterm or at term, and the number of PTBs was insufficient for detailed analyses. Studies in Nicaragua [28] showed that cortisol predicted low birthweight and small for gestational age (described below), but not PTB.

Anxiety, Depression, and Common Mental Disorders: Four of ten studies showed significant relationships. Studies of 550 women in Iran [34] showed that Depression Anxiety Stress Scales scores at 24–32 weeks pregnancy predicted gestational age (β = -0.18). Studies of 600 women in Iran [38] showed that State-Trait Anxiety Inventory scores were higher among women with PTB compared to term deliveries, and that state anxiety scores predicted PTB (RR = 3.1, CI = 2.05–4.7). One study of 865 women in Brazil [27] showed that General Health Questionnaire scores >3 at 30–36 weeks predicted PTB (RR = 2.32, CI = 1.18–4.60), controlling for confounders. In contrast, Perceived Stress Scale (discussed above) and State-Trait Anxiety Inventory scores were not significant predictors. Finally, case-control studies of 959 women in Peru [39] showed that Patient Health Questionnaire and Depression Anxiety Stress Scales scores predicted PTB, with ORs for the moderate-severe symptomatic group ranging from 2.76 for the anxiety subscale (CI = 1.83–4.16) to 11.07 (CI = 5.64–21.71) for stress.

Six other studies showed no significant relationships. Studies of 719 women in Ghana and Cote d’Ivoire [40] showed that 3^rd^T Patient Health Questionnaire and Generalized Anxiety Disorder Scale scores did not predict gestational age or PTB. The sample represented uncomplicated pregnancies; thus, the authors suggest that results might highlight confounding effects of pregnancy complications in the relationship between maternal depression and anxiety, and birth outcomes. Studies of 726 women from South Africa [41] showed that depression, based on Beck Depression Inventory scores at 20–28 weeks pregnancy, did not predict PTB, although relationships with other outcomes are discussed below.

Of studies showing no significant relationships, two are limited by sample size. Studies of Hospital Anxiety and Depression Scale scores among 463 women in China [42] showed that PTB risk did not differ between the Symptom and Symptomless groups. However, only 13 women (4.2%) delivered preterm, all in the symptomless group. Similarly, case-control studies of 125 women from Pakistan [30] showed no relationships between depression, based on the Center for Epidemiological Studies Depression Scale, and PTB (OR = 1.4, p = 0.697), but the small number of preterm births (n = 15) limits analyses. Finally, two studies with large samples might benefit from more detailed analyses. Studies among 737 women in Sri Lanka [35] showed no relationships between General Health Questionnaire scores and PTB. Scores were dichotomized; more detailed analyses might be possible. Similarly, studies of 600 women from Pakistan [43] showed that prevalence of “emotional stress” based on the Edinburgh Postnatal Depression Scale was 69% among cases who delivered preterm and 55% among controls (p<0.01), but the cutoffs were not well described, and stress did not predict PTB in logistic regressions including covariates.

Other stress measures: Three final studies of PTB assessed occupational stress [44], trauma [45], and pregnancy-related stress [46], with mixed results. Case-control studies among 2189 women in China [46] showed that high stress based on the Pregnancy Stress Rating Scale predicted PTB (RR = 2.92, CI = 1.12–7.58), but low and medium levels were not significant predictors. Studies among 2623 women in Mexico [44] showed that occupational stress did not predict PTB, but highlighted other occupational risk factors. Finally, among 544 women in South Africa [45], prevalence of childhood (34%) and lifetime trauma (67%) was high, but trauma did not predict PTB. However, trauma predicted infant head circumference, discussed below.

#### Birthweight, low birthweight, and small for gestational age

Twenty-one studies (44%) analyzed birthweight, low birthweight (LBW), and small for gestational age. Fourteen (67%) of these showed significant associations with at least one stress measure.

Perceived stress: Two of three studies showed significant relationships. Studies of Perceived Stress Scale scores among 474 Romanian in a smoking cessation program [25], discussed above with PTB and gestational age, showed that high stress predicted a 113g reduction in birthweight (CI = -213–-11). Studies in Nicaragua [28] showed that interviewer-assessed perceived stress predicted increased cortisol, which then predicted LBW and small for gestational age. These results are discussed more in the section on cortisol. In contrast, studies of 46 women from Columbia [29] analyzing periodontitis and Perceived Stress Scale scores found that perceived stress did not predict PTB. However, the small sample limits the analyses.

Life events: Two of four studies showed significant relationships. Studies of 1800 women in China [33] showed that life events during the 1^st^T, but not 2^nd^T or 3^rd^T, predicted birthweight (RR = -122.97, CI = -166.64–-79.29), even adjusting for covariates. Similarly, studies of 1197 women in South Africa [47] used the Social Readjustment Rating Scale to assess life events in the year before delivery. Results in one cohort showed that greater life events scores predicted lower birthweight. Another study of 726 women in South Africa [41] showed no direct effects of life events on birthweight, but highlighted relationships between life events and antenatal depression. Finally, studies of 1065 women in Ethiopia [48] showed no relationships between life events and birthweight.

Cortisol: Three of four studies showed significant relationships. Studies of 1041 women from Bangladesh [37] demonstrated that higher 3^rd^T cortisol predicted smaller birthweight among male but not female infants. Studies of 1391 women from Malawi [26] showed that greater cortisol at 28 and 36 weeks pregnancy predicted lower BW, particularly among primiparous women. Studies of 147 women in Nicaragua [28] showed that low social resources and high perceived stress predicted increased cortisol, and that increased cortisol then predicted LBW and small for gestational age. In contrast, studies of 737 women in a nutrient supplement study from Nepal [36] showed that 3^rd^T cortisol did not predict birthweight (*β* = 4.2, p = 0.17) or LBW (OR = 0.98, CI = 0.95–1.01).

Anxiety, Depression, and Common Mental Disorders: Six of ten studies showed significant relationships. These all had relatively large samples and considered covariates in analyses. Studies of 600 women in Iran [38] showed that high state anxiety (State-Trait Anxiety Inventory score ≥45) predicted LBW (RR = 2.6, CI = 1.6–4.2). Studies assessing 3^rd^T Edinburgh Postnatal Depression Scale and State-Trait Anxiety Inventory scores among 583 mothers in Bangladesh [49] showed that depressive symptoms (OR = 2.24, CI = 1.37–3.68) and anxiety (OR = 2.08, CI = 1.30–3.25) predicted LBW. Studies of 865 women in Brazil [27] showed that General Health Questionnaire scores >3 at 30–36 weeks pregnancy predicted LBW (RR = 1.97, CI = 1.12–3.47). Similarly, case-control studies of 835 women in Sri Lanka [50] showed that 2^nd^T General Health Questionnaire scores predicted small for gestational age (OR = 1.92, CI = 1.17–3.14). Studies of 537 women in Ethiopia [51] showed that depressive symptoms assessed with the Edinburgh Postnatal Depression Scale predicted LBW (OR = 1.87, CI = 1.09–3.21). Finally, studies of 726 women in South Africa [41] showed that depression, based on the Beck Depression Inventory at 20–28 weeks pregnancy, predicted weight for age Z-scores (OR = 0.2, CI = 0.02–0.4), although relationships did not persist when controlling for life events.

Four studies (including three discussed above with gestational age and PTB) failed to show significant relationships. Studies of 719 women with healthy, uncomplicated pregnancies in Ghana and Cote d’Ivoire [40] showed that 3^rd^T Patient Health Questionnaire and Generalized Anxiety Disorder Scale scores did not predict birthweight. Similarly, studies of 521 women in Ethiopia [48] showed that 3^rd^T Self-Reporting Questionnaire scores did not predict BW. Studies of 175 women in Thailand who completed the Center for Epidemiological Studies Depression Scale at 26–38 weeks pregnancy showed no main effects of antenatal depression on BW [52]. Finally, studies of 463 women in China [42] showed no associations between Hospital Anxiety and Depression Scale scores and birthweight but, as noted above, analyses suffer from small sample sizes.

Other stress measures: Other studies of birthweight, LBW, and small for gestational age assessed occupational stress, trauma, and intimate partner violence. Two studies of occupational stress showed significant effects. Studies among 2623 women in Mexico [44] showed that conflicts at work predicted small for gestational age, but only among women who delivered at the public assistance hospital, which typically sees the poorest women (OR = 4.93, CI = 2.09–11.66). Studies of 792 women working at a chemical plant in China [53] showed interactive effects of benzene exposure and work stress. Adjusted mean birthweight was 183g lower among women with both work stress and benzene exposure compared to those with neither. Three studies of trauma and intimate partner violence showed no direct effects, but highlighted other important relationships. Studies of 726 women from South Africa [41] showed that childhood and past-year trauma predicted antenatal depression, but did not directly predict weight for age Z-scores. Similarly, among 544 women in South Africa [45], prevalence of childhood (34%) and lifetime trauma (67%) were high, but did not predict weight outcomes. Finally, studies of 147 women from Nicaragua [28] demonstrated that intimate partner violence during pregnancy predicted cortisol, which then predicted lower BW. Intimate partner violence might affect both physical and psychosocial health, which complicates analyses. However, the study from Nicaragua [28] highlights maternal stress as a potential mechanism through which intimate partner violence influences birthweight.

#### Other infant outcomes at birth

Anthropometric measurements: Six studies analyzed other anthropometric measures at birth, including head circumference, head circumference Z-scores, head circumference for age Z-scores, birth length, and length for age Z-scores. Five showed significant results. Studies of 726 women from South Africa [41] showed that antenatal depression, based on Beck Depression Inventory scores at 20–28 weeks pregnancy, predicted smaller head circumference for age Z-scores (OR = 0.3, CI = 0.1–0.6). Similarly, among 544 women in South Africa [45], lifetime trauma exposure predicted a 0.3 unit reduction in head circumference for age Z-scores (p = 0.026). Studies of 1391 women from Malawi [26] showed that perceived stress at 28 and 36 weeks pregnancy predicted shorter length for age Z-scores, but not head circumference Z-scores. In contrast, studies of 461 women in China [42] showed that head circumference and birth length did not differ among infants of women symptomatic and asymptomatic for anxiety and depression. Finally, studies of 719 women in Ghana and Cote d’Ivoire [40] showed that mean head circumference did not differ among low-obstetric risk women with and without anxiety and depression in the 3^rd^T, although more detailed analyses are needed.

Two studies analyzed relationships between these measures and cortisol. Studies of 1041 women from Bangladesh [37] demonstrated that higher 3^rd^T cortisol predicted smaller head circumference among male but not female infants, mirroring results for birthweight (discussed above). In contrast, studies of 1391 women from Malawi [26] (discussed above) showed that cortisol at 28 and 36 weeks pregnancy did not predict length for age Z-scores or head circumference Z-scores.

Apgar scores: Studies in Iran [54] of 29 pregnant women with anxiety or depression assigned to a stress management intervention (n = 14) or control group (n = 15) showed that mean 1-minute Apgar scores were higher in the intervention (8.93) than control (8.07) group (p = 0.01), with similar results at five minutes. Two other previously-described studies also assessed Apgar scores. Studies of 719 women in Ghana and Cote d’Ivoire [40] showed that Apgar scores were weakly correlated with Patient Health Questionnaire (r = -0.106, p<0.05) and Generalized Anxiety Disorder Scale scores (r = -0.102, p<0.05). In contrast, studies of 461 women in China [42] showed that Apgar scores did not differ among those who were symptomatic or asymptomatic for anxiety and depression.

#### Other outcomes of the pregnancy

Six articles examined other outcomes of the pregnancy or at birth (three of these also analyzed other birth outcomes, discussed above). Studies of 1065 pregnant women in Ethiopia [48] showed no associations between stillbirth or neonatal death and 3^rd^T common mental disorder symptoms, life events, or worry in pregnancy. However, common mental disorder symptoms were associated with prolonged labor (Low symptoms, RR = 1.4, CI = 1.0–1.9; High, RR = 1.6, CI = 1.0–2.6). Similarly, analyses of 461 women in China [42] showed that prevalence of prolonged pregnancy was higher among women symptomatic for anxiety and depression (n = 3, 8.3%) compared to symptomless women (n = 5, 1.6%) (RR = 4.08, CI = 1.25–13.33), although several other outcomes did not differ among groups.

Two studies assessed miscarriage, with one showing significant results. Studies of 22 women in rural Guatemala [55] followed in detail from before pregnancy showed that increased urinary cortisol predicted pregnancy loss (OR = 2.7, CI = 1.2–6.2), and highlighted that placentation might be a particularly sensitive period to maternal stressors. In contrast, case-control studies of 33 women in Malaysia [56] showed no differences in mean morning cortisol at 24–32 weeks pregnancy between women with a history of PTB and miscarriage, and those with no such history. However, it is unclear how many women were assigned to the case group because of previous miscarriages, PTB, or both.

Finally, two studies assessed methylation patterns following exposure to severe stress and trauma during pregnancy. Studies of 24 mother-infant dyads in Democratic Republic of Congo [57] show differential methylation of maternal venous blood, placental tissue, and umbilical cord blood among women exposed to chronic stress and war trauma. Correcting for multiple testing, four CpG sites were differentially methylated in at least one tissue, all associated with war trauma. Methylation at four sites predicted birthweight, collectively explaining 55% of variance. Studies by the same group [58] of 25 women in Democratic Republic of Congo showed that BW was correlated with maternal deprivation (r = -0.484), mundane stress (r = -0.521), and war stress (r = -0.620). Of the war stressors, rape was the most important predictor, explaining 31% of variance. War stress was correlated with birthweight (r = -0.449), and with cord blood methylation of the NR3C1 gene (r = 0.586), which might have long-term effects on the child’s stress response.

#### Outcomes later in infancy and childhood

Among the 48 studies, 14 (29%) analyzed outcomes later in infancy (n = 5) and childhood (n = 9). These are grouped by age period and outcome below rather than stress measure because of the limited number of studies.

Infancy–Temperament: Three studies assessed temperament, all showing significant results. Studies of 100 women in India [59] showed no linear relationships between 3^rd^T General Health Questionnaire scores and temperament at 1–4 months of age. However, adaptability and approach scores were higher among infants whose mothers had low (score <7) compared to high distress. Studies of 378 women in Vietnam [60] showed that 2^nd^T Edinburgh Postnatal Depression Scale scores predicted Bayley Scales of Infant Development social-emotional scores at age 6 months (β = -3.30, CI = -6.32–-2.89). Path analyses showed that antenatal common mental disorders predicted postnatal common mental disorders, which then predicted social-emotional scores. Studies in China of 38 women with healthy, uncomplicated pregnancies [61] compared Bayley and Toddler Temperament Scale scores at age 16 and 18 months among infants of 38 mothers exposed to 1^st^T life events with 114 unexposed controls. Controlling for covariates, exposure to life events predicted lower infant mental development scores, and higher regularity and persistence and attention span scores (on which lower scores indicate better responses).

Infancy–Motor Development: The study of 38 women in China discussed above [61] showed no relationships between maternal life events and infant motor development. Others showed mixed results. Studies in South Africa [62] categorized women into low and high stress groups based on feelings about pregnancy, life events, and family relationships, and analyzed infants’ neuromotor development shortly after birth, and at 1 and 3 months. Motor scores did not differ between groups. However, excluding babies with birthweight >8 pounds (n = 35), significant differences were noted: 42.5% of infants in the high maternal stress group had motor scores <3, compared to 15.4% in the low-stress group. The limited analyses restrict generalization, and the exclusion of larger babies is not clearly justified. However, the rigorous assessments of neuromotor development are an important contribution to the literature.

Infancy–Illness: Studies in Ethiopia [63] analyzed 3^rd^T and postpartum Self-Reporting Questionnaire scores among 954 women, and maternal-reported infant illness at age 2 months. Multivariate analyses showed that persistent (during both pregnancy and postpartum) common mental disorder symptoms predicted infant diarrhoea (RR = 2.15, CI = 1.39–3.34). Relationships with other illnesses were inconsistent.

Childhood–Behavioral Outcomes: Six studies analyzed behavioral and psychiatric outcomes, with five showing significant relationships. Studies in Nicaragua [64] analyzed 2^nd^T–3^rd^T salivary cortisol and Self-Reporting Questionnaire scores among 147 women. Children were assessed at age 9 using the Child Behavior Checklist, and their cortisol was analyzed. Maternal cortisol predicted total Child Behavior Checklist scores (r = 0.31), and internalizing (r = 0.28) and externalizing symptoms (r = 0.35), but not children’s cortisol. Self-Reporting Questionnaire scores did not predict any child outcomes. Researchers in Brazil [65] assessed discord during pregnancy using a single retrospective interview question, and Attention Deficit Hyperactivity Disorder (ADHD) symptoms among 370 children age 6–13 with the Child Behavior Checklist and Teacher Report Form. Discord during pregnancy predicted mother-reported ADHD (OR = 4.54, CI = 2.16–9.57), controlling for covariates. Similarly, studies of 241 women in Thailand [66] assessed emotional distress during pregnancy using a single retrospective question, and compared results between 122 6-12-year-old children with ADHD, and 119 controls with no behavioral problems. Maternal distress predicted ADHD in multivariate analyses (OR = 2.99, CI = 1.43–5.40). Studies in South Africa [67] assessed 3^rd^T exposure to 16 stressors, divided into high and low exposure, among 953 women, and children’s behavioral problems at ages 2 and 4 using the Behaviour Screening Questionnaire. Controlling for covariates, maternal stress exposure predicted behavioral problems among children at age 4 (OR = 2.66, CI = 1.28–5.54). Finally, researchers in Brazil [68] used a single questionnaire item, administered shortly after delivery, to assess mood symptoms during pregnancy. Psychiatric disorders were assessed among 3581 children at age 6 using the Development and Well-Being Assessment. Controlling for confounders, maternal mood symptoms predicted psychiatric disorders among children (OR = 1.97, CI = 1.60–2.41). In contrast, studies in Turkey [69] retrospectively assessed stressors experienced during pregnancy using a tailor-made form and showed no differences in exposure between 105 3-5-year-old children with psychiatric complaints, and 205 controls.

Childhood–Other Outcomes: Other studies analyzed heart rate [70], physical growth [71], and asthma [72]. Studies in China analyzed depression and anxiety among 216 pregnant women [70], and children’s heart rate and blood pressure before, during, and after a video game stressor at age 7–9. Heart rate and blood pressure stress responses were higher among children of mothers with anxiety, and anxiety predicted all heart rate and blood pressure outcomes in multivariate analyses. Results for depression were significant but less consistent. Studies in Brazil [71] analyzed body mass index Z-scores at age 5–8 among 409 children whose mothers completed the Perceived Stress Scale, General Health Questionnaire, and State-Trait Anxiety Inventory three times during pregnancy. In multivariate regressions, 2^nd^T General Health Questionnaire scores predicted children’s body mass index Z-scores (β = -0.09). Finally, studies in Mexico [72] analyzed relationships between 2^nd^T–3^rd^T life events and child asthma symptoms at age 4. Controlling for confounders, negative life events during pregnancy predicted ever wheeze among children (RR = 1.08, CI = 1.00–1.16) and wheeze in the past 12 months (RR = 1.12, CI = 1.00–1.26).

## Discussion

This scoping review highlights a number of patterns in prenatal stress research already observed in other studies. For example, two-thirds of studies that assessed relationships between prenatal stress and birthweight showed modest but significant associations, consistent with the general patterns observed in past research from mostly high-income countries [1, 12]. Similarly, effects vary based on timing of exposure, which has been widely observed in other studies [5, 9, 10, 73]. Effects were evident even later in childhood, consistent with long-term programming effects of prenatal stress observed by others [2–4, 8–10].

The synthesis also highlights wide variability that has already been observed in the literature. For example, relationships between life events and birthweight were not significant in two of four studies, which likely reflects variability in the number and type of life events assessed, complications with retrospective assessment in many studies, and confounding by other variables, as in past research from mostly high-income countries [1]. Similarly, relationships between prenatal stress and gestational age were significant in only about half of studies. Researchers in high-income countries have observed wide heterogeneity in relationships between prenatal stress and gestational age, reflecting variability in relationships based on the timing of stress exposure during pregnancy, lack of distinction between the particular components of the stress response that might predict gestational age, and interactive relationships with other factors such as maternal infection or nutrition [73]. In particular, gestational age seems to be more strongly linked to prenatal anxiety than to stress or depression [74], and results might be masked in studies that cannot distinguish between these constructs.

Finally, our synthesis highlights broader challenges to research on prenatal stress and child development in LMICs. For example, although many studies from high-income countries have shown relationships between perceived stress or common mental disorders and gestational age [1, 12, 73], results here were significant in only one of six studies assessing perceived stress, and only four of ten studies assessing anxiety, depression, and common mental disorders. The interacting effects of confounding variables might be a particularly important source of heterogeneity in this context. Furthermore, measurement of gestational age is a challenge in many LMICs where women might not receive early prenatal care [75]. Thus, inconsistencies in relationships between prenatal stress and gestational age or preterm birth might be further exacerbated by methodological challenges. Similarly, as noted above, distinguishing between constructs such as stress, distress, anxiety, and depression is important, but this might not be possible in LMICs lacking detailed evaluation tools. Some studies specifically compared effects of stress, anxiety, and depression on developmental outcomes [39], but others focused more on similarities between the constructs of stress and common mental disorders in the context. For example, authors noted that family life stressors [49] and stress due to poverty or ill health [40] might exacerbate or underlie part of the relationship between common mental disorders and perinatal outcomes, and thus discussed these concepts together rather than distinguishing between them.

Synthesis of these studies highlights other important steps for future research. First, existing research is biased toward upper-middle income countries (60% of studies). In particular, three upper-middle income countries (China, South Africa, and Brazil) accounted for 16 of 48 studies reviewed (33%). Studies in low income countries [26, 36, 48, 51, 57, 58, 63] represented only 15%, and lower-middle income [28, 30, 35, 37, 40, 43, 49, 50, 55, 59, 60, 64] only 25%. This likely reflects limited research and healthcare infrastructure that complicates data collection and follow-up. However, our reviews show that, in general, researchers were able to collect sufficient data during pregnancy and up to birth to conduct solid analyses of relationships. Of the 19 studies in low and lower-middle income countries, 12 [26, 28, 36, 37, 49–51, 55, 57, 58, 60, 64] showed significant relationships. Four others [40, 48, 59, 63] were less robust, but showed relationships with some outcomes. Although low, lower-middle, and upper-middle income countries share many similar challenges in maternal, infant, and psychosocial health, studies from diverse regions remain necessary because of differences in public health burdens such as communicable diseases, maternal and perinatal conditions and nutritional deficiencies [76] and infant mortality [77], as well as different public health spending patterns [15], maternal care services [75], and intervention priorities [78]. These factors might particularly affect the application of prenatal stress research findings to healthcare. More research in low and lower-middle income countries is warranted to foster generalization of major findings and their eventual application to public health.

This review also indicates a bias for studies of outcomes at birth, with limited follow-up beyond, across all three income groups. Although studies of relationships between prenatal stress and birth outcomes remain necessary, longer-term follow-up is essential to assess how patterns seen at birth might translate into child and adult well-being, and to identify relationships with outcomes not measurable at birth. Limited infrastructure likely hinders long-term follow-up of mothers and infants in LMICs.

Assessment of the varied stress measures employed shows the applicability of many instruments used in high-income countries across all three income groups, but also the usefulness of and need for tailored measures. Existing surveys of stress, anxiety, depression, and life events were used in 32 studies, including four from low income countries [26, 48, 51, 63], nine from lower-middle income [30, 35, 40, 43, 49, 50, 59, 60, 64], and 19 from upper-middle income countries [25, 27, 29, 33, 34, 38, 39, 41, 42, 44–47, 52, 54, 61, 70–72]. Cortisol was analyzed in eight, including two from low-income countries [26, 36], five from lower-middle income [28, 30, 37, 55, 64], and one from upper-middle income [56]. The use of these existing measures facilitates comparisons across studies. However, collecting biological samples is not always feasible where infrastructure is limited, and existing questionnaires might be difficult to employ because of a lack of validated translations, high illiteracy rates, and because women might not be familiar with self-report surveys. Thus, tailor-made questionnaires are sometimes the only option. Five studies [31, 32, 62, 67, 69] used tailor-made surveys to assess life events and perceived stress. Two of these [32, 69] do not describe the instruments in detail, but the others provide enough information to allow basic comparisons across studies. Three others [28, 57, 58] used ethnographic interviews with structured questionnaires to assess trauma and violence. Although this limits generalization, the authors highlight that culturally sensitive interview methods might allow for more complete and accurate ascertainment of stressors than existing or unfamiliar questionnaires [57], and the development of rapport with the interviewer might help to minimize underreporting [28]. Finally, four studies [53, 65, 66, 68] used responses to a single questionnaire item to assess stress, all showing significant results. While this again limits generalization, it demonstrates the usefulness of simple measures that might pave the way for more detailed future evaluations.

These studies also highlighted the importance of the broader social and environmental context in studies of prenatal stress in LMICs. For example, in one study [62], women’s reports of difficulties predicted outcomes, but results were less consistent for situations that might objectively appear stressful such as poor diet or household crowding, highlighting the importance of women’s own perceptions of stress. Similarly, researchers suggested that the lack of relationship between common mental disorders and birth outcomes in one study [48] might reflect interrelationships among factors such as illness, undernutrition, and socioeconomic disadvantage that were common in the population and might overwhelm the effects of common mental disorders. Qualitative interviews [79] from the same group indicated that worry about the delivery was an important source of distress, reflecting the reality of high maternal and infant morbidity and mortality in the region. Other themes highlighted in articles reviewed here such as unwanted pregnancy and intimate partner violence are not unique to LMICs, but lack of resources in these settings might exacerbate women’s associated distress.

The conclusions that can be drawn from several studies are limited because of small sample size and limited statistical analyses, which was observed across the three income categories. Twelve studies with particularly limited samples and analyses were identified (see Discussion points in S2 Table and S2 Appendix), and several others would benefit from more rigorous assessment of covariates. Stress during pregnancy is associated with numerous covariates such as maternal age, socioeconomic status, nutrition, marital status, physical health, and pregnancy history that might all affect outcomes independently or in interaction with stress, but these were not considered in many studies. However, careful consideration of these variables might highlight pathways underlying relationships between prenatal stress and child development. Three studies used path analyses [28, 34, 60] to address such relationships; these methods might be particularly important for guiding future interventions. Studies of interactive effects should also be prioritized. For example, research in Bangladesh [37] showed interactive effects of stress and nutrition, suggesting that early nutrient supplementation might ameliorate the negative effects of prenatal stress on infant growth. When exposure to stress cannot be changed, other interacting variables might provide targets to alleviate its effects. More rigorous assessment of interactions and pathways is needed to identify these targets.

### Strengths and limitations

This is a broad study aiming to synthesize available research related to prenatal stress and child development from LMICs. The inclusion of multiple measures of stress and developmental outcomes resulted in a large body of literature for analysis, allowing us to highlight the wide diversity in prenatal stress research in LMICs. However, given the breadth of studies included, with important differences in samples, predictor measures, and outcomes, calculation and direct comparison of effect sizes was not conducted. Furthermore, detailed comparison of study results from LMICs and high income countries is beyond the scope of this review. Direct comparison of the direction and strength of relationships from LMICs and high income countries represents an important area for future reviews focusing on specific stress measures, such as life events or perceived stress, or developmental outcomes, such as low birthweight or motor development.

### Conclusions

Many current studies show relationships between prenatal stress and outcomes of the pregnancy, infant, or child in LMICs. Synthesis of these reviews highlights the applicability in LMICs of methods used in high-income countries, but also the need for tailored tools adapted to the cultural context. These should include careful assessment of covariates, especially those that might have interactive effects with maternal stress. Studies including longer-term follow-up should be prioritized. The development and validation of brief and simple survey measures that could be incorporated into broader studies of maternal and child health might be an important avenue to guide future research. Direct comparison of studies analyzing specific stress measures or developmental outcomes from LMICs and high income countries represents an important area for future review.

## Supporting information

S1 Table Search strategy and country list. (DOCX)

S2 Table Summary of studies of prenatal stress and pregnancy and child health outcomes in LMICs: Details and discussion points. (DOCX)

S1 Appendix PRISMA checklist. (DOC)

S2 Appendix Assessment of methodological quality. (DOCX)

## Abbreviations

1^st^T, 2^nd^T, 3^rd^T: first, second, and third trimester
ADHD: Attention Deficit Hyperactivity Disorder
BL: Birth length
BMI: Body Mass Index (kg/m^2^)
BMIZ: Body mass index Z-score
BP: Blood pressure
BW: Birth weight
CBCL: Child Behavior Checklist
CI: Confidence interval
CMD: Common Mental Disorder
DASS: Depression Anxiety Stress Scales
DSM: Diagnostic and Statistical Manual of Mental Disorders
GA: Gestational age
GHQ: General Health Questionnaire
HC: Head circumference
HCAZ: Head circumference for age Z-score
HCZ: Head circumference Z-score
HR: Heart rate
IUGR: Intra-uterine growth restriction
LAZ: Length for age Z-score
LBW: Low birthweight
LM income: Lower-middle income
LMIC: Low- and middle-income countries
MLEI: Modified Life Events Inventory
OR: Odds ratio
PHQ: Patient Health Questionnaire
PSS: Perceived Stress Scale
PTB: Preterm birth
RR: Relative risk
SGA: Small for gestational age
SRQ: Self-Reporting Questionnaire
SRRS: Social Readjustment Rating Scale
STAI: State-Trait Anxiety Inventory
TTS: Toddler Temperament Scale
UM income: Upper-middle income
WAZ: Weight for age Z-score
