# Supplementary material for: Prenatal stress and child development: A scoping review of research in low- and middle-income countries
Source: PLoS One. 2018 Dec 28;13(12):e0207235. doi: 10.1371/journal.pone.0207235 (PMC6310253; doi:10.1371/journal.pone.0207235)
Supplement: S1 Table — (DOCX) [file pone.0207235.s001.docx]

**S1 Table:** Search strategy and country list

| **Database** | **Search Strategy** |
| --- | --- |
| PubMed | Search was performed using MeSH Terms as follows: "Humans"[Mesh] AND ("Stress, Psychological"[Mesh] OR "Stress, Physiological"[Mesh] OR "Disasters"[Mesh] OR "Life Change Events"[Mesh]) AND "Prenatal Exposure Delayed Effects"[Mesh] AND ("Developing Countries"[Mesh] as well as the list of all LMIC countries searched as keywords. |
| Scopus | Search terms included those related to pregnancy (TITLE-ABS (( prenatal OR maternal OR mother* OR perinatal OR peripartum OR antenatal OR antepartum OR gestation* OR trimester OR pregnan* OR intrauterine OR "in utero" ) W/3 ( stress* OR adversity OR distress* OR anxiety ))); those related to infant or child outcomes (TITLE-ABS ( outcomes OR "birth weight" OR "birth length" OR "birth size" OR "live birth" OR "still birth" OR stillbirth OR "growth retardation" OR "head circumference" OR apgar OR "preterm birth" OR "pre-term birth" OR "preterm delivery" OR "pre-term delivery" OR "preterm labor" OR "pre-term labor" OR "preterm labour" OR "pre-term labour" OR "shorter gestation" OR "length of gestation" OR "gestational age" OR ( ( infant OR baby OR child* OR fetal OR foetal OR offspring ) W/5 development* ) OR ( ( infant OR baby OR child* OR fetal OR foetal OR offspring ) W/5 neurodevelopment* ) OR ( ( infant OR baby OR child* OR fetal OR foetal OR offspring ) W/5 growth ) OR ( health W/3 ( infant OR baby OR child* OR fetal OR foetal OR offspring ) ) OR prematur* OR "pregnancy loss" OR "disease risk" OR dohad OR "development* program*" OR "fetal program*" OR "foetal program*" OR "prenatal program*" OR "delayed effects" OR "late effects" OR "lifetime effects" OR "lifetime costs" OR "long-term effects" ))) and those related to developing countries or LMICs (TITLE-ABS-KEY ( "developing country" OR "developing nation" OR ( ( "least developed" OR "less developed" OR "under developed" ) PRE/2 country ) OR ( ( "least developed" OR "less developed" OR "under developed" ) PRE/2 nation ) OR lmic OR ( ( "low income" OR "lower-middle income" OR "upper-middle income" ) PRE/2 country ) OR ( ( "low income" OR "lower-middle income" OR "upper-middle income" ) PRE/2 nation ) OR ( "third world" PRE/2 ( country OR nation ))) as well as the list of all countries classified by the World Bank as low income (Gross National Income per capita (GNI) < $1,025 USD), lower-middle income (GNI $1,026-$4,035), or upper-middle income (GNI $4,036-$12,475 and names of their inhabitants (ex: Brazilian, Ethiopian, Malaysian) |
| PsycINFO | All proximity operators (PRE/n and W/n) of the Scopus request were replaced by AND operator to avoid search engine overflow. |

| Low income countries | Lower-middle income countries | Upper-middle income countries |
| --- | --- | --- |
| Afghanistan | Armenia | Albania |
| Benin | Bangladesh | Algeria |
| Burkina Faso | Bhutan | American Samoa |
| Burundi | Bolivia | Angola |
| Central African Rep. | Cabo Verde | Argentina |
| Chad | Cambodia | Azerbaijan |
| Comoros | Cameroon | Belarus |
| Congo, Dem. Rep. | Congo, Rep. | Belize |
| Eritrea | Côte d'Ivoire | Bosnia and Herzegovina |
| Ethiopia | Djibouti | Botswana |
| Gambia, The | Egypt, Arab Rep. | Brazil |
| Guinea | El Salvador | Bulgaria |
| Guinea-Bissau | Ghana | China |
| Haiti | Guatemala | Colombia |
| Korea, Dem. Rep. | Honduras | Costa Rica |
| Liberia | India | Cuba |
| Madagascar | Indonesia | Dominica |
| Malawi | Kenya | Dominican Republic |
| Mali | Kiribati | Ecuador |
| Mozambique | Kosovo | Equatorial Guinea |
| Nepal | Kyrgyz Rep. | Fiji |
| Niger | Lao PDR | Gabon |
| Rwanda | Lesotho | Georgia |
| Senegal | Mauritania | Grenada |
| Sierra Leone | Micronesia, Fed. Sts. | Guyana |
| Somalia | Moldova | Iran, Islamic Rep. |
| South Sudan | Mongolia | Iraq |
| Tanzania | Morocco | Jamaica |
| Togo | Myanmar | Jordan |
| Uganda | Nicaragua | Kazakhstan |
| Zimbabwe | Nigeria | Lebanon |
|  | Pakistan | Libya |
|  | Papua New Guinea | Macedonia, FYR |
|  | Philippines | Malaysia |
|  | Samoa | Maldives |
|  | São Tomé and Principe | Marshall Islands |
|  | Solomon Islands | Mauritius |
|  | Sri Lanka | Mexico |
|  | Sudan | Montenegro |
|  | Swaziland | Namibia |
|  | Syrian Arab Republic | Palau |
|  | Tajikistan | Panama |
|  | Timor-Leste | Paraguay |
|  | Tonga | Peru |
|  | Tunisia | Romania |
|  | Ukraine | Russian Federation |
|  | Uzbekistan | Serbia |
|  | Vanuatu | South Africa |
|  | Vietnam | St. Lucia |
|  | West Bank and Gaza | St. Vincent and the Grenadines |
|  | Yemen, Rep. | Suriname |
|  | Zambia | Thailand |
|  |  | Turkey |
|  |  | Turkmenistan |
|  |  | Tuvalu |
|  |  | Venezuela, RB |
