# Supplementary material for: Prenatal stress and child development: A scoping review of research in low- and middle-income countries
Source: PLoS One. 2018 Dec 28;13(12):e0207235. doi: 10.1371/journal.pone.0207235 (PMC6310253; doi:10.1371/journal.pone.0207235)
Supplement: S2 Table — (DOCX) [file pone.0207235.s002.docx]

**S2 Table:** Summary of studies of prenatal stress and pregnancy and child health outcomes in LMICs: details and discussion points

| Article | Country | Sample | Stress measure | Other predictors /covariates | Outcome measures | Results | Discussion/Notes |
| --- | --- | --- | --- | --- | --- | --- | --- |
| Abeysena et al. 2010 (35) | Sri Lanka  (South Asia, lower-middle income) | 737 women age≥15 with uncomplicated pregnancies and no pre-existing diabetes, hyper-tension, epilepsy, or major psychiatric conditions, recruited at <16 wks pregnancy in antenatal clinics from May 2001–Apr 2002 | Modified Life Events Inventory (MLEI) and General Health Questionnaire (GHQ). Scores were dichotomized for analyses; cutoffs were GHQ>5, and MLEI≥2. | Sociodemographic characteristics;  occupational and environmental exposures, including heat, noise, radiation, chemicals; time spent walking, sitting, standing, and sleeping at work and home | PTB  GA was calculated based on ultrasound scan around 20 wks of pregnancy and LMP | Univariate logistic regression indicated that GHQ and MLEI scores were not significant predictors of PTB. Multivariate analyses indicated a trend for exposure to MLEIs during the 2^nd^T and PTB (p=0.09, OR 1.80). | Discussing the trend-level relationship between 2^nd^T MLEIs and PTB, the authors calculated a power of 36%, reflecting inadequate sample size when analyzing results by trimester. They suggest further research on trimester-specific effects. |
| Abramson et al. 1961 (62) | South Africa (Sub-Saharan Africa, upper-middle income): poor suburb of Durban | 101 pregnant Indian women ages 16-46 who resided in the area from 1959-1960, and their babies. Initial examinations were conducted on 84 infants. | In the 3^rd^T, women responded to 15 interview questions addressing feelings about pregnancy, life events, and relationships with husband and in-laws. Stress was categorized from 1 (minimal) to 5 (extremely marked). Analyses compared babies of women with “low stress” (1-2) and “high stress” (3-5). | Sociodemographic characteristics;  maternal nutritional status (based on skin lesions, skinfold thickness, and height and weight)  Infant length was measured at the first assessment, and weight at every assessment | Neuromotor development: Researchers visited the home on average 3, 32, and 93 days after delivery.  5 tests of gross motor behaviour were conducted and results summed into a final motor score. | Excluding babies with BW>8.0 pounds, 42.5% in the high-stress group had motor scores <3, compared to 15.4% in the low-stress group. Results were similar at the 2^nd^ but not 3^rd^ evaluation. High-stress women were older, less educated, from more crowded and poor homes, and had inferior nutritional status. | Women’s reports of difficulties predicted outcomes, but results were less consistent for situations that might objectively appear to be stressful (e.g. household crowding, poor diet), highlighting the importance of women’s own perceptions. Results must be interpreted cautiously due to the small sample and limited analyses. |
| Arffin et al. 2012 (56) | Malaysia (East Asia and Pacific, upper-middle income): Kuala Lampur | 33 multiparous pregnant women (20 with history of miscarriage and PTB, 13 controls) ages 23-39 recruited at 24-32 wks pregnancy from the antenatal clinic. Exclusion criteria included smoking (active or passive), drug abuse, medical conditions, and multiple fetuses. | Salivary cortisol, collected before 12:00 p.m. from 24-32 wks of pregnancy | Salivary cotinine; questionnaire measures of medical and pregnancy history, age, occupation, ethnicity, body weight | History of miscarriages and PTB, assessed from medical records | Mean salivary cortisol levels did not differ significantly between the test (1.016±SEM 0.182 lg/ml) and control (0.978±0.298 lg/ml) groups (p=0.392). | Details such as the number of previous preterm births or miscarriages are not provided, and important covariates are not measured or described. The large range in time of day and gestation wks complicates the cortisol analyses and, together with sample size, might account for the lack of significant results. |
| Arteaga-Guerra et al. 2010 (29) | Colombia  (Latin America & the Caribbean, upper-middle income): Pasto, Nariño | 46 women ages 18-34 who had a vaginal birth, recruited within 48 hours after delivery at the local hospital from Jun 2008–Apr 2009. Women were excluded if they smoked, consumed alcohol “in a great amount,” took drugs, did not attend prenatal visits, had “other medical diseases”, or less than junior high education. | Perceived Stress Scale (PSS-10), administered 48 hours postpartum | Periodontitis, identified by two dental clinicians based on buccal and lingual dental plaque and gingival bleeding. | PTB, LBW, PTB & LBW combined (PLBW).  GA was based on LMP or results of 1^st^T ultrasound | Perceived stress was not associated with PTB or LBW. The combination of periodontitis but no elevated stress predicted PLBW (OR=10.3, CI=1.1, 93.2, p=0.01). | The authors suggest that stress might act as a protective factor for PLBW by mediating inflammation induced by periodontitis, and that low perceived stress might reflect an inadequate and harmful response to high levels of daily stress in the context. The small sample and division into groups of 9 (+periodontitis, +stress), 27 (+, -), 2 (-, +), and 10 (-, -) limits analyses. |
| Baig et al. 2013 (43) | Pakistan  (South Asia, lower-middle income): Karachi | 600 women with live spontaneous preterm (Cases, n=300) or term (Controls, n=300) births with no congenital anomalies, matched for parity and SES, who delivered in the Civil Hospital and Abbasi Shaheed Hospital from Jan–May 2011 | Edinburgh post-natal depression scale.  Participants were defined as having emotional stress during pregnancy or not; the cutoff used was not described. | Blood haemoglobin levels; questionnaire of SES and pregnancy risk factors (history of PTB, hypertension, diabetes, urogenital tract infections, periodontal disease, parity), as well as weight, diet, and drug addiction during pregnancy | PTB  GA was based on LMP or early ultrasound (before 20 wks) | Prevalence of emotional stress was 69% among cases and 55% among controls (p<0.01). In logistic regression, risk factors for PTB included maternal weight <50 kg, periodontal diseases, low haemoglobin, history of PTB, and poor nutritional status. | The authors suggest that stress increased risk of PTB, presumably based on differences in emotional stress prevalence between cases and controls. Stress was not identified as a risk factor in logistic regression analyses. More detailed analyses are needed. |
| Barrios et al. 2014 (31) | Peru  (Latin America & the Caribbean, upper-middle income): Lima | 959 women with singleton spontaneous preterm (Cases, n=479) or term (Controls, n=480) births with no malformations. Participants were recruited by daily monitoring at 3 hospitals in Lima from Jan 2009–Jul 2010. | Life events during pregnancy, including death of a close family member, divorce or separation, job change, financial trouble, fights and arguments with partner, and attendance at the wedding of a close friend or relative, collected via interview. | Questionnaires of demographics and SES, cigarette and alcohol use, and parity  Review of medical records to assess reproductive history, blood pressure, and obstetric complications | PTB  GA was calculated based on LMP and confirmed by early ultrasound (before 20 wks) | In logistic regression analyses controlling for covariates, PTB was associated with severe life events including death of a relative (OR=2.10, CI=1.38, 3.20), divorce or separation (OR=2.09, CI=1.10, 4.00), financial troubles (OR=2.70, CI=1.85, 3.94), and fights with partner (OR=2.40, CI=1.78, 3.17). Risk increased with # of life events. | The authors note that some studies indicate significant effects of life events only during particular trimesters, whereas effects were significant in this study without considering timing. Limitations include retrospective data collection and potential reverse causality (ex: women with difficult pregnancies might be forced to leave work). |
| Başgül et al. 2011 (69) | Turkey (Europe and Central Asia, upper-middle income): Izmit and Kocaeli, urban and rural areas | 309 children age 3-5 yrs: 204 randomly sampled from primary healthcare units from Feb–Apr 2006, and 105 with “various psychiatric complaints” who consulted the Kocaeli Univ. Pediatric Psychiatry Dept. from Mar–Sept 2006; and their parents | Stressors experienced by the mother were assessed using an investigator-prepared form during assessments of natal and postnatal characteristics. No details on the questions are provided. | Sociodemographic data form: demographics of the child and parent, family structure, consanguinity, siblings, “natal and  postnatal characteristics and developmental  features of the child,” type of housing | The Early Childhood Inventory-4: Parent Form was used to evaluate behavioral, emotional, and cognitive problems in children.  Families were interviewed based on DSM-IV, and the child was observed in free play. | At least one stress factor was reported in 50% of mothers. No differences in stress or other predictor variables were found between children with psychiatric conditions and those without.  Financial difficulties and problems with the husband or in-laws were the most common stress factors reported. | Although the stress variable did not differ among groups, the authors suggest that “the increased incidence of psychiatric disorders in children of mothers who reported stress… emphasized the importance of avoiding stress during this period”. More detailed analyses are needed. |
| Bhat et al. 2015 (59) | India (South Asia, lower-middle income): Bangalore | 100 women with normal singleton pregnancies attending routine antenatal checkup at St. John’s Medical College Hospital. Women with preterm or low birthweight deliveries, pregnancy-induced hypertension or gestational diabetes, use of alcohol/drugs or smoking, and blood borne diseases were excluded. | Maternal prenatal psychological distress, assessed with the GHQ-28 during the 3^rd^T  Scores ≥7 were used as a cutoff in some analyses. | Maternal age, education, marital and employment status, mode of delivery.  Postpartum GHQ-28 (completed by 85 women)  Infant salivary cortisol at 1-4 months of age | Temperament (9 dimensions) at 1-4 months of age, rated by the mothers using the Early Infancy Temperament Questionnaire  BW, GA, and Apgar score were discussed, but not in detail. | Controlling for confounders, GHQ scores were not correlated with any temperament dimension. In univariate analyses, mean adaptability and approach scores were higher among mothers with GHQ<7 (3.11 and 2.70) compared to those with GHQ≥7 (2.67, 2.06) (p=0.03, p=0.05). Results persisted in multivariate analyses. Other temperament dimensions did not differ between groups. | The higher adaptability and approach scores among mothers with GHQ scores <7 might suggest that their infants have, or are perceived to have, an easier temperament. The authors note that relationships between prenatal stress and development might be curvilinear such that moderate levels may be optimal, underlying nonsignificant results. Cultural factors such as high levels of social support from extended family might also affect the results. |
| Bindt et al. 2013 (40) | Ghana  (Sub-Saharan Africa, lower-middle income)  Cote d’Ivoire  (Sub-Saharan Africa, lower-middle income) | 719 pregnant women assessed during 3^rd^T antenatal visits at Komfo Anokye Hospital (Ghana) and Abobo Community Hospital (Côte d’Ivoire) from Mar 2010–Dec 2011. Analyses were limited to low-risk women (age≥18, singleton pregnancy; no chronic physical disease, diabetes,  hypertension, or preeclampsia) | Depression symptoms and anxiety over the past 2 wks were assessed with the Patient Health Questionnaire and the 7-item Generalized Anxiety Disorder scale, respectively. Scores ≥10 were used as the cutoff to categorize depression and anxiety. | Data on previous pregnancy complications, maternal age, infections, malaria treatment, and caesarian section were collected (methods not detailed). An SES score was constructed based on ownership of refrigerator, car, and bed net, toilet type, and education. Women’s hemoglobin level and height were measured. | Birth outcomes: BW, LBW, head circumference, GA and PTB, 5-minute Apgar score (<7 defined as “low”).  GA was based on  2^nd^T ultrasound | Anxiety and depression scores were weakly correlated with Apgar scores (r=-0.106 and -0.102, respectively) but not with BW or GA.  Depression and anxiety were not predictive of BW or PTB in univariate or multivariate analyses.  Authors note that mean HC at delivery did not differ between groups, but detailed analyses are not provided. | Previous studies in these countries show prevalence of LBW and PTB of 9% and 17%, whereas prevalence in this sample was 1.7% and 4.2%. Because the sample was low-risk, the authors suggest that results might highlight a confounding effect of pregnancy complications in the relationship between maternal depression and anxiety, and birth outcomes. |
| Brittain et al. 2015 (41) | South Africa  (Sub-Saharan Africa, upper-middle income): Western Cape | 726 pregnant women (20-28 wks gestation) ≥age 18 from two health clinics in a peri-urban, low-SES community | - Beck Depression Inventory (BDI-II); scores ≥20 categorized as antenatal depression  - World Mental Health Life Events Questionnaire, to assess stressful life events in the last 12 months  - Modified Posttraumatic Stress Disorder (PTSD) symptom scale | - Questionnaire to assess pregnancy planning and intimate partner violence (IPV) in the past 12 months  - Childhood Trauma Questionnaire  - Alcohol, Smoking and Substance Involvement Screening Test  - SES score based on employment, education, income, and assets | BW and HC; WAZ, HCAZ, and SGA based on Fenton charts  GA based on 2^nd^T ultrasound, symphysis-fundal height, or LMP | 15% of births were preterm and 26% were SGA. No associations were observed between antenatal depression and PTB. Controlling for SES and clinic, antenatal depression predicted smaller WAZ (OR=0.2, CI=0.02, 0.4) and HCAZ (OR=0.3, CI=0.1, 0.6).  Relationships between depression and WAZ did not persist when controlling for stressful life events. | Prevalence of depression (21%), IPV (33%), childhood trauma (20%), and suspected PTSD (18%) were high. Risk of depression was associated with single status, unemployment, low SES, stressful life events, unplanned pregnancy, childhood trauma, IPV, and suspected PTSD. Women in many communities face similar risk factors, highlighting the need for further studies in LMICs. |
| Cerón-Mireles et al. 1996 (44) | Mexico (Latin America & the Caribbean, upper-middle income): Mexico City | 2623 women who gave birth to singleton infants with no congenital anomalies, and who worked at least 3 months during pregnancy. All mothers who gave birth at 3 participating hospitals were screened for eligibility. Of 9549 deliveries, 2767 mothers had been employed at least 3 months during pregnancy. 2663 of these women were interviewed in the hospital | Occupational stress was measured with Karasek's Job Content Questionnaire. Women who scored above the median for demands and below the median for control were considered to have high-strain jobs. An index similar to that of Mamelle was used to assess occupational fatigue. | Interviews were used to assess demographics, reproductive history, smoking, self-reported height and weight, prenatal care utilization, and social support. Self-esteem was measured using a scale in the Nat. Inst. For Occupational Safety and Health General Job Stress Questionnaire. Medical records were reviewed for pregnancy complications. | Birth outcomes collected from medical records, including GA and PTB, BW and SGA (≤10th percentile  Based on BW-for-GA distributions in Mexican infants by Jurado et al. (1970).  GA was based on LMP. | In multivariate analyses, risk factors for SGA included working >50 hrs/week, standing >7 hrs/day, and having no antenatal leave. Conflicts at work predicted risk only among women who delivered at the public assistance hospital, which typically sees the poorest women (OR=4.93, CI=2.09, 11.66). PTB risk factors included having no antenatal leave benefits, and taking <3 wks of leave. | Occupational stressors might be less important risk factors for adverse birth outcomes in the population than length of workday and posture. However, poor women might be vulnerable to conflicts at work. Scales used to assess occupational stress in industrialized countries did not predict LBW or PTB in this population.  Enforcement of labor laws and paid leave benefits might provide routes to reduce adverse birth outcomes. |
| Chen et al. 2000 (53) | China  (East Asia and Pacific, upper-middle income): Beijing | 792 women who worked at Beijing Yanshan Petro-chemical Corp., had a single live birth at the staff hospital from May 1996–Dec 1998, and were exposed to benzene (n=354) or not exposed (n=438) during pregnancy. Women exposed to solvents other than benzene, or who smoked or drank alcohol during pregnancy, were excluded. | Perceived work stress was assessed by questionnaire using a single question, “How stressed do you feel at your workplace? (1=no or low, 2=moderate,  3=high).” Responses were dichotomized into "low" and "moderate or high" for analyses. | Questionnaires were used to obtain data on maternal demographics, cigarette smoking, alcohol consumption, diet, physical activity, occupational exposure, and medical and reproductive history. Clinical data including pre-pregnancy weight and height were recorded by a trained nurse. | BW  BW was measured in the delivery room and recorded from medical records by a trained nurse. GA was based on LMP. Note that because all births are planned due to the need to apply for birth permission in China, dates for LMP are considered highly reliable in the setting. | Mutivariate regression indicated interactive effects of benzene exposure and work stress. Adjusted mean BW was 3445g among the group with neither work stress nor benzene exposure, 3426g with work stress alone, 3430g with benzene exposure alone, and 3262g with both exposures. | Exposure to organic solvents is common in the general population and, in interaction work stress, might contribute to LBW. Research among women working with organic solvents should consider stress, as well. The single question on work stress has been used in other studies, and has been shown to predict abnormal duration of the menstrual cycle [80] and frequency of dysmenorrhea [81] |
| Christian et al. 2016 (36) | Nepal (South Asia, low income): Sarlahi | 737 pregnant women who provided blood samples in the 1^stT^ and 3^rd^T. Women of reproductive age were visited every 5 wks to complete a urine pregnancy test. Women were assigned to one of 5 supplement groups including vitamin A (control) plus: 1) folic acid, 2) folic acid + iron, 3) folic acid + iron + zinc, 4) folic acid + iron + zinc + other micronutrients | 3^rd^T serum cortisol | A baseline interview was used to assess date of LMP, diet, morbidity and work histories in the previous 7 days, SES, and reproductive history. Serum erythropoietin (EPO) was measured in the 3^rd^T. | GA, LBW, and PTB  GA was based on LMP queried at the baseline interview, and veriﬁed alongside prospectively collected data on the menstrual cycle and the date of the positive pregnancy test. | Controlling for EPO, supplement group, and gestational age at blood collection, cortisol predicted PTB (OR=1.04, CI=1.00, 1.08), but not BW (*β* = 4.2, p=0.17*)* or LBW (OR=0.98, CI = 0.95, 1.01)  Cortisol was marginally lower by 1.32 μg/dL  (p=0.062) in the multiple micronutrient supplement group relative to controls, and did not differ in other groups. | Iron and multiple micronutrient supplementation may enhance birth outcomes by reducing maternal cortisol. Programs for multiple micronutrient supplementation might be an important addition to existing policies for iron-folic acid supplementation to improve birth outcomes in low income settings. |
| Fan et al. 2016 (70) | China  (East Asia and Pacific, upper-middle income) | 216 mothers who were assessed for anxiety and depression during the 1^st^T (n=71), 2^nd^T (n=72), or 3^rd^T (n=73), and their children. Women with a history of psychiatric or cardiovascular disease were excluded. | - Hamilton Anxiety Scale: Scores were summed and anxiety classified as mild (18-25), moderate (26-30), and severe (>30)  - Hamilton Rating Scale for Depression  (HRSD): Scores were summed and depression classified as mild (10-19), moderate (20-29), or severe (>30) | Children’s fasting glucose and BMI are mentioned in the results, but their measurement is not described. | Resting blood pressure (BP) and heart rate (HR) at age 7-9 years. Children played video games for 10 minutes. BP and HR were measured before (rest), during (stress), and after (recovery). Maximum increases in HR and BP between each period were analyzed. | HR and BP stress responses were higher among children whose mothers had mild to severe anxiety compared to none. Results for depression were less consistent, although recovery BP varied among groups. Anxiety predicted all HR and BP measurements in multivariate analyses. Depression predicted resting HR, stress SBP, and recovery SBP and DBP. | The authors highlight potential confounding of maternal mood postpartum, and genetic transmission of susceptibility. Results contribute to our knowledge of maternal anxiety and children’s stress response, but more detailed statistical analyses controlling for covariates are necessary to strengthen conclusions. |
| Frith et al. 2015 (37) | Bangladesh  (South Asia, lower-middle income) | 1041 pregnant women in “Maternal and  Infant Nutritional Interventions, Matlab” trial who gave birth between Jun 2003–Mar 2004 and for whom cortisol samples were available. The RCT assigned women to “early start” for daily food supplementation (~9 wks gestation) or “usual start” (~20 wks gestation). | Morning salivary cortisol (30 min to 1 h post-awakening) at 28-32 wks gestation. Concentrations were categorized based on the median value of 9.6 nmol/l as lower (<9.6) or higher  (>9.6) for descriptive analyses. | Questionnaire measures of parity, age and wealth index during early pregnancy based on land ownership, characteristics of the household dwelling, and ownership of durables. Maternal height and weight were measured at 8–10 wks of pregnancy. | BW, birth length (BL), head circumference (HC), GA  GA was based on LMP, which was queried monthly by community health workers, who visited women and offered them a pregnancy if their LMP was overdue. | Male (but not female) infants of mothers with higher cortisol had smaller BW, HC, and GA. In general linear models, relationships between cortisol and BW and HC differed by supplementation group. In the “usual start” group, greater cortisol predicted smaller values. In the “early start” group, cortisol did not predict outcomes. Results were unchanged by covariates. | Results highlight a difference in BW of ~280 g between male infants of women with the lowest and highest cortisol values in the “usual start” group.  Early food supplementation might alleviate negative effects of stress on infant growth. Where LBW is a public health concern, policies to provide food supplements in the 1^st^T might improve birth outcomes. |
| Hanlon et al. 2009 (48) | Ethiopia (Sub-Saharan Africa, low income):  Butajira Rural Health Programme | 1065 pregnant women in the 3^rd^T from Jul 2005 – Feb 2006; 521 singleton infants whose BW was measured within 48 hours after birth. See also [63] | - Self-Reporting Questionnaire (SRQ-20) to assess Common Mental Disorders (CMD) in the preceding month, categorized as no symptoms (SRQ=0), low (SRQ=1-5), and high (SRQ≥6)  - List of Threatening Experiences questionnaire (stressful life events)  - Women were asked whether they worried about the upcoming delivery. | SES (hunger in the preceding month,  perceptions of wealth relative to others, indebtedness); education, marital status; parity, previous stillbirth or neonatal death; use of alcohol, khat and tobacco; antenatal care; tetanus immunization, physical assault, fever, and malaria during pregnancy; unwanted pregnancy; maternal anthropometrics | BW, stillbirth, and neonatal mortality (within 28 days of birth); self-reported prolonged  labor (≥24 h) and time to initiation of breast-feeding (within an hour of birth, 1 – 8 h,  8 – 24 h, >24 h) | Controlling for confounders, there were no associations between BW, stillbirth, or neonatal death and CMD symptoms, stressful life events, or worry in pregnancy.  Prolonged labor was associated with CMD symptoms (Low, RR=1.4, CI=1.0, 1.9; High, RR=1.6, CI=1.0, 2.6) and worry about the delivery  (RR=1.5, CI=1.0, 2.1). Analyses did not support a linear effect of SRQ score. | The authors suggest that interrelationships among factors such as under-nutrition, physical ill-health, socioeconomic disadvantage and lack of education might overwhelm any additional effects of CMD on birthweight and underlie the lack of significant relationships. |
| Isaksson et al. 2015 (64) | Nicaragua  (Latin America & the Caribbean, lower-middle income) | 147 women participating in a prospective study of the effects of partner violence on gestation length and fetal growth, who contributed saliva samples during the 2^nd^ or 3^rd^T. Nine years later, 70 mothers and their children were recruited for follow-up | - Morning and afternoon salivary cortisol during pregnancy and nine years later  - The Self Reporting Questionnaire (SRQ-20). Women with scores >6 were categorized as distressed | Interviews to assess SES (housing materials,  school enrollment of children, # of unemployed persons  in the household, piped water, toilet, latrine)  - Abuse against women, questionnaire developed by the WHO Multi-Country Study on Women’s Health and Domestic Violence Against Women | Child's psychiatric symptoms at age 9 years using the Child Behavior  Checklist (CBCL)  Child’s salivary cortisol | Morning cortisol during pregnancy was associated with total CBCL scores  (r=0.31, p=0.009), and with internalizing (r=0.28, p=0.020) and externalizing symptoms (r=0.35, p=0.003). Results persisted when adjusting for confounders. Cortisol during pregnancy did not correlate with children’s cortisol at age 9. SRQ was not associated with CBCL scores or children’s cortisol. | The authors note that the HPA axis responds to psychosocial but also physiological stressors. This may obscure relationships between maternal psychological stressors and children’s cortisol outcomes. The combined effects of both psychosocial and physiological stressors on cortisol measures might be particularly important in low SES communities where food insecurity might cause physiological stress. |
| Karamoozian and Askarizaden 2015 (54) | Iran (Middle East and North Africa, upper-middle income): Kerman | 29 2^nd^T pregnant women with anxiety or depression assigned to a control group (n=15, no intervention) or to a cognitive-behavioral stress management (CBSM) intervention (n=14). Women were primiparous with low-risk pregnancies, at least high school education, no history of chronic physical or mental disease, and were not currently treated for depression or anxiety. | Edinburgh Postnatal Depression Scale (EPDS) and Pregnancy-Related Anxiety Questionnaire (PRAQ) were completed before the intervention. The experimental group received 12 weekly CBSM sessions. EPDS and PRAQ were completed again after the intervention. | None discussed | Apgar scores at 1 and 5 minutes | Anxiety and depression scores differed between groups after treatment, indicating that the CBSM reduced anxiety (effect size=0.57) and depression (effect size=0.51). Mean one-minute Apgar scores were higher in the experimental (8.93) than control (8.07) group (p=0.01); similar results were observed at 5 minutes (9.71 and 9.27, respectively; p=0.05). | This study shows that a stress management program during pregnancy reduced anxiety and depression. The program, which including teaching participants to recognize symptoms of stress, and to implement relaxation techniques, was feasible in the setting. Generalization of results is difficult due to the small sample of symptomatic women. Analyses would benefit from inclusion of covariates. |
| Kertes et al. 2016 (57) | Democratic Republic of Congo (Sub-Saharan Africa, low income) | 24 mother-newborn dyads at HEAL Africa hospital in Goma, interviewed  within 24 hours of birth | Semi-structured ethnographic interviews were used to assess stressful events during pregnancy. 32 items were identified reflecting chronic stress (socioemotional e.g. unhappy marriage, crying during pregnancy, no help with domestic chores; and SES, e.g. not owning home, trouble paying bills, past food insufficiency) or war-related trauma (e.g., rape, refugee status, family member killed). | Assessment of confounders is not described, but maternal age, parity, and smoking status are mentioned in the results | DNA samples were isolated on site within several hours of delivery from maternal venous blood, placental tissue, and umbilical cord blood. Methylation levels were determined at multiple CpGs in four HPA axis-related genes: CRH, CRHBP, NR3C1, and FKBP5.  BW was collected at delivery. | 18 CpG sites were significantly associated with either chronic stress (n=11), war trauma (n=14), or both (n=6). Correcting for multiple testing, 8 CpG sites remained, all associated with war trauma.  Methylation levels at four CpG sites, situated at transcription factor binding sites in NR3C1 and CRH, collectively explained 55% of the variance in BW. | This study is among the first to examine relationships between prenatal stress and epigenetic outcomes in a developing country. The women in this sample faced extreme chronic stress and trauma. This likely enhanced the power to detect biological effects, but might also limit generalization.  The authors note the importance of culturally sensitive ethnographic interviews in assessing stressors. |
| Koen et al. 2016 (45) | South Africa (Sub-Saharan Africa, upper-middle income): Cape Town (poor-peri-urban community) | 544 women ≥age 18 and their infants (n=546) recruited from two primary health care clinics into the Drakenstein Child Health Study (DCHS) from Mar 2012–Oct 2014 at 20 to 28 wks gestation. Women who did not attend postnatal care clinics or who  intended to move out of the district within 2 years after the infant’s birth were not eligible | - Childhood Trauma  Questionnaire (CTQ)  - Intimate  Partner Violence (IPV) Questionnaire (adapted from WHO multicountry study)  - Mini International Neuropsychiatric Interview  (MINI), used to assess lifetime post-traumatic stress disorder (PTSD) | - Demographics, including SES based on education, employment, income, assets, and market access  - Alcohol, Smoking, and Substance Involvement  Screening Test (ASSIST)  - World Mental Health Life Events Questionnaire  - Beck Depression Inventory  - Edinburgh Postnatal Depression Scale (EPDS)  - SRQ-20 | BW, HC, and GA at birth, used to calculate WAZ, HCAZ, SGA, and PTB  GA was based on 2^nd^T ultrasound, fundal height, or LMP | PTSD did not predict WAZ, HCAZ, SGA, or PTB. Adjusting for covariates including IPV and recent life events, lifetime trauma exposure (MINI) predicted a 0.3 unit reduction in HCAZ (p=0.026). Results persisted at a trend level when controlling for WAZ (p=0.054). Multivariate results for other outcomes are not presented, but the Discussion notes that trauma did not predict most outcomes. | Prevalence of childhood trauma (34%), IPV (32%), and lifetime trauma (67%) were high. Trauma predicted reduced HCAZ, controlling for a number of covariates. The authors suggest that detection and treatment of trauma in the pre- and peripartum period might help to curb detrimental effects on mother and child. The authors suggest focusing on mediators of the association between maternal trauma and adverse birth outcomes such as substance use and psychosocial stress. |
| Meghea et al. 2014 (25) | Romania (Europe & Central Asia, upper-middle income): urban sample | 474 pregnant women ≥age 18 who were enrolled in the Smoking during Pregnancy in Romania (SPRO) project, and who were followed-up by phone after birth. SPRO recruited women who sought antenatal care or were confined in bed because of a difficult pregnancy in the two largest obstetrics clinics in Cluj-Napoca,  Romania, from Nov 2008–Aug 2009. | Perceived Stress Scale-4. A binary "high stress" indicator was defined based on a score ≥4, the median sample value. | Questionnaires were used to assess maternal demographics, whether the pregnancy was wanted, and parity. The Romanian translation of the Patient Health Questionnaire-2  (PHQ-2) was used to assess depressive symptoms. | BW, GA, SGA, PTB  BW and GA were self-reported during the follow-up phone interview. SGA was defined as BW<10th percentile. | 61.9% of smokers, 50.1% of non-smokers, and 51.9% of women who quit smoking during pregnancy had high stress (p=0.28). In multivariate linear regression including smoking status and other covariates, high stress predicted a 113g reduction in BW (CI=-213, -11). In similar logistic regression models, high stress predicted PTB (OR=2.81, CI=1.17, 6.76). | Results highlighted that high stress during pregnancy was associated with BW and PTB independent of smoking and other risk factors. The authors suggest that smoking cessation efforts in Romania should integrate psychological components to address antenatal stress. |
| Mirabzadeh et al. 2013 (34) | Iran (Middle East and North Africa, upper-middle income): Tehran | 550 pregnant women (singleton pregnancies, 24-32 wks gestation) recruited from Jun 2012-Feb 2013 from one public hospital in each of four geographical zones. Women with known medical problems before and during pregnancy, and a history of or current pregnancy complications were excluded. | - DASS-21 Questionnaire, administered at recruitment, categorized as mild (scores 1-7), moderate (8-14), and severe (15-21)  - Stressful Life Events Questionnaire (Holms and Rahe, 1963) | - Questionnaires to assess demographics, reproductive history  - Multidimensional Scale of Perceived Social Support, categorized as low (scores 12-48), moderate (49-68), and high (69-84) | PTB  GA was based on LMP or ultrasound | GA was negatively correlated with depression, anxiety, stress, and DASS scores. Path analysis showed direct relationships among DASS and GA (β=-0.18). Social support and stressful life events were associated with DASS scores (β=-0.26 and β=0.22, respectively) and thereby indirectly related to PTB. Risk was higher among women with low SES. | Among multiple variables analyzed, stress, depression and anxiety had the greatest direct effects on PTB. SES and social support moderated this relationship. The inclusion of path analyses is unique; these can provide particularly important insights to guide future research. |
| Mulligan et al. 2012 (58) | Democratic Republic of Congo (Sub-Saharan Africa, low income) | 25 women who delivered their babies at HEAL Africa hospital in Goma from Jul–Aug 2010 | - Detailed ethnographic interviews of deprivation (e.g. owning a home, material possessions), “mundane stressors” (e.g. pregnancy-related stress, food insecurity, intimate partner violence, illnesses), war stressors (e.g. rape, refugee status,)  - Trauma surveys adapted from the Peritraumatic Distress Inventory | Ethnographic interviews  were used to assess reproductive history, past traumatic exposures, and  general health history, but specific variables are not described | Genomic DNA was extracted from 50 maternal venous blood and umbilical cord blood samples. Methylation levels of 39 sites in the NR3C1 promoter were assayed.  BW was analyzed, but its measurement is not described. | BW was correlated with maternal deprivation (r=-0.484), mundane stress (r=-0.521), and war stress (r=-0.620). Of the war stressors, rape was the most important predictor, explaining 31% of variance. War stress was correlated with cord blood methylation levels of the NR3C1 gene (r=0.586) and BW (r=-0.449). Relationships between war stress and maternal methylation, and between maternal methylation and BW, were not significant. | These studies support a relationship of an environmental stressor (war-related stress) affecting phenotype (BW) through epigenetic changes (methylation).  Methylation changes in NR3C1 might have long-term effects on the child’s potential to adapt to stressors later in life through reduced plasticity in stress-related genes, and thereby increase his or her risk of chronic psychosocial health disorders. |
| Nasiri et al. 2010 (38) | Iran (Middle East and North Africa, upper-middle income): Babol | 600 women ages 18-45 with singleton pregnancies, recruited at 20-28 wks of pregnancy from Sep 2004-May 2006 in a prospective cohort study. Exclusion criteria included chronic diseases, psychological disorders, previous PTB, other pregnancy complications, and “bad events during the last 3 months”. | Spielberger State-Trait Anxiety Inventory (STAI). A cut-off of ≥45 was used to categorize state and trait anxiety. | Questionnaires were used to assess sociodemographic characteristics, parity, pregnancy weight gain, antenatal care, conditions of conception (natural, medically assisted, contraception failure), vaginal bleeding, infections | PTB and LBW  Gestational age was based on ultrasound before 20 wks (usually at 12 to 18 wks). | Mean state and trait anxiety scores were higher among women with PTB (42.7 and 52.9, respectively) compared to term pregnancies (37.8 and 50.7), and women with LBW (42.3 and 52.9) compared to no LBW (38.1 and 50.1). State anxiety score ≥45 was associated with PTB  (RR=3.1, 95% CI= 2.05, 4.7) and LBW (RR=2.6, CI=1.6, 4.2). | Although most control variables did not differ among women with and without state anxiety, the study would benefit from more advanced analyses to control for confounders. Nevertheless, results highlight that anxiety during pregnancy is predicts PTB and LBW. The authors suggest the need for therapeutic measures among women with anxiety to help prevent LBW and PTB. |
| Nasreen et al. 2010 (49) | Bangladesh  (South Asia, lower-middle income) | 583 mothers of singleton, term infants, studied from the 3^rd^T to 6-8 months postpartum. Women came from an original cohort of 720 consecutive women from 154 villages who participated in data collection from Jul 2008-Aug 2009. Exclusion criteria for the original study were emigration from the study area, intrauterine death, or abortion. | Edinburgh Postnatal Depressive Scale: A cutoff of ≥10 was used to categorize depression.  State Trait Anxiety Inventory: A cutoff of ≥46 was used to categorize anxiety. | - SES: education, landholding, daily food expenditure  - Women’s weight, height, BMI, and mid upper arm circumference (MUAC)  - Number of children and antenatal consultations  - Social support: family structure (e.g. nuclear or extended family), physical and psychological support, and physical violence. Variables were dichotomized for analyses. | LBW  Other obstetric outcomes were assessed, including length of pregnancy, complications during labor, live or still birth, and infant BL and HC. However, these were not the focus of the study, and only basic descriptive analyses of these outcomes are discussed. | Prevalence of depression was 8%, and trait anxiety 26%. 19% of babies were LBW.  Controlling for covariates, multiple logistic regression analyses indicated that depressive symptoms predicted LBW (OR=2.24, CI=1.37, 3.68). Results were similar for anxiety (OR=2.08, CI=1.30, 3.25). Other risk factors included low SES, low MUAC, and less psycho-logical support. | LBW is a main cause of infant morbidity and mortality in Bangladesh, and maternal nutrition is a key risk factor. This study shows that depression and anxiety predict LBW independent of nutritional status (MUAC). The authors suggest that poor nutritional status is not necessarily a result of poverty but of maternal mental disorders, and thus might affect women even in food-sufficient regions. The importance of policies to detect maternal depression and anxiety is emphasized. |
| Nepomnaschy et al. 2006 (55) | Guatemala  (Latin America & the Caribbean, lower-middle income): rural Kaqchikel Mayan community in the southwestern highlands | 61 women ages 18-34 who were not pregnant at the onset of the study, were cohabitating with their husband, had parity ≥1, not using contraception, and last birth >6 months before the onset of the study. Over the course of 12 mos, 22 conceptions were detected. Of these, 9 were carried to term and 13 were lost. | First morning urine specimens were collected 3x/wk, and concentrations of cortisol and other hormones assayed. Timing of ovulation, chemical pregnancy, and pregnancy loss was identified. Pregnancies in which the average  standardized cortisol following conception was higher than baseline were classified as exposed to increased cortisol. | To reduce the risk of confounders, participants were asked to collect their urine samples as soon as they woke up each morning, before they consumed food or performed physical activity. Samples collected after eating or beginning chores were discarded. None of the participants smoked or consumed alcohol. | Pregnancy: successful or unsuccessful | Mean cortisol levels were higher in unsuccessful than in successful pregnancies. Increased cortisol predicted pregnancy loss (OR=2.7, CI=1.2, 6.2). 90% of the “increased cortisol” pregnancies but only 33% of the “normal cortisol” pregnancies were lost. Furthermore, unsuccessful pregnancies presented a larger proportion of cortisol peaks than successful ones. | Results are contextualized in terms of evolutionary theories of costs of reproduction under impoverished reproductive conditions. Fluctuations in cortisol might serve as a physiological cue that conditions for reproduction are deteriorating. Effects are detectable in early pregnancy but not later in gestation, suggesting that the placentation period might be a sensitive period for maternal stress. |
| Pires et al. 2013 (65) | Brazil  (Latin America & the Caribbean, upper-middle income): Rio de Janeiro | 370 children ages 6-13 participating in a 2005 longitudinal study measuring risk factors for ADHD. A random sampling plan identified students from clusters of classrooms and schools. Parents were invited to participate in interviews at the schools. | Mothers were asked “whether the pregnancy was a peaceful time for the mother or was marked by discord and arguments” | Questionnaires were used to assess maternal education; whether the mother was a restless child; social support; family functioning (McMaster Family Assessment Device); life events in the past year; and whether mothers used alcohol, tobacco, or drugs while pregnant  Child’s IQ was assessed using the Wechsler Intelligence Scale for Children | Child Behavior Checklist (CBCL) and the Teacher Report Form (TRF) assessing children’s ADHD symptoms | In univariate analyses, discord during pregnancy predicted mother-reported ADHD (OR=4.54, CI=  2.16, 9.57). Results persisted in multi-variate analyses. Discord during pregnancy did not predict teacher reported ADHD.  Other predictors for mother-reported ADHD included family functioning, social support, and life events in the past year. Child IQ was the most important predictor of teacher-reported ADHD. | The study uses only a single measure of discord during pregnancy, collected retrospectively, that limits the conclusions that can be drawn. However, results highlight a potential link between the prenatal environment and ADHD risk that might inform more detailed analyses in future studies in this setting and other LMICs. |
| Qiao et al. 2012 (42) | China  (East Asia and Pacific, upper-middle income): Shanghai | 463 women ages 18-45, at 20-41 wks pregnancy, attending ultrasound exams at one of 4 hospitals affiliated with Shanghai Tongji Univ. from Feb–Sept 2008. Exclusion criteria included history of severe mental disorders, nervous system diseases, hypertension, diabetes, cardiac diseases, liver and renal inadequacy, and use of antidepressants during pregnancy. | Hospital Anxiety and Depression Scale (HADS). A cutoff of ≥9 was used to group women into the “Symptom” group | Questionnaire of demographic information (age, education, occupation, religion, marital status) and “obstetric and gynecological data” | Pregnancy length (preterm, term, prolonged); induction of labour; placental abruption; placenta praevia; multiple pregnancy; fetal macrosomia; amniotic fluid amount; abnormal development of fetus; fetal position; fetal distress; delivery method; abnormal labor; labor complications; neonatal data: BW, BL, gender, Apgar scores | 11% of women were in the Symptom group. Covariates did not differ among groups. Prevalence of prolonged pregnancy was higher in the Symptom (n=3, 8.3%) than in the Symptomless group (n=5, 1.6%) (RR=4.08, CI=1.25, 13.33). Other obstetric and neonatal outcomes did not differ among groups. | The use of only chi-square analyses and the small number of women in most outcome categories limits the conclusions that can be drawn. Thus, the authors’ conclusion that “neonatal outcome does not deteriorate, despite the women's impaired mental health during pregnancy” must be considered cautiously. However, the study includes many outcomes that are not assessed in other studies, and that might be useful in guiding future research. |
| Qu et al. 2016 (46) | China  (East Asia and Pacific, upper-middle income): First Affiliated Hospital of Sum Yat-sen University and Guangzhou Women and Children’s Medical Center | 2189 Han Chinese pregnant women (3^rd^T) ≥age 20 who completed antenatal examinations at one of the two study hospitals, who completed questionnaires between Mar 2011-Mar 2012 and their infants, including 130 premature (cases) and 2059 term (control) deliveries. Women with history of adverse pregnancy outcomes, medical and obstetric complications, and who became pregnant by assisted reproductive technology were excluded. | Revised Pregnancy Stress Rating Scale. Scores were categorized as 0 (no stress), >0-1 (low stress), >1-2 (medium stress), >2-3 (high stress) | - Questionnaire measures of sociodemographic characteristics, pre-pregnancy BMI  - Coping Style Questionnaire. Scores were categorized as low, medium-low, medium-high, and high coping based on quartiles. Higher scores represent a better coping style.  - Social Support Scale. Scores were categorized as low, medium-low, medium-high, and high support based on quartiles. Higher scores represent a better social support. | PTB | In multiple logistic regression adjusting for sociodemographic confounders, BMI, coping style, and social support, high pregnancy specific stress predicted PTB (RR=2.92, CI=1.12, 7.58). Low and medium levels of pregnancy-specific stress were not significant predictors of PTB. | The authors note that the study is limited by the small number of women in each stress group: the high-stress group included only 79 women and 10 PTBs. Thus, results must be interpreted cautiously. The assessment of social support and coping styles strengthens the methodology. The authors suggest in the Discussion that these were significant predictors of PTB, although results are not detailed. Greater discussion of these variables might guide future research in similar settings. |
| Ramchandani et al. 2010 (67) | South Africa  (Sub-Saharan Africa, upper-middle income) : Soweto-Johannesburg | 953 mothers (primarily socially disadvantaged) of singleton children born in 1990, who completed stress questionnaires. Children were born during a 7-week period characterized by volatility and political violence. Participants were drawn from the Birth to Twenty cohort study of physical and psychosocial development of urban children. | Interviews were conducted in the 3^rd^T to assess 16 stressors including marital stress (partner violence, relationship breakdown), family stress (fight with family, family member with drug problem or disability), economic stress, and societal stress and violence (in danger of being killed, witness to a violent crime). Scores were summed and a cutoff ≥4 was categorized as high stress. | Maternal demographics: housing, ownership of assets (television,  car, refrigerator, washing machine, telephone), medical insurance coverage, ethnicity, marital status, age, education, smoking, and alcohol use during pregnancy  Pitt Depression Questionnaire was administered 6 months postpartum. A cutoff ≥20 was used to categorize probable depression.  Child BW and GA were recorded from birth records. | Richman Behaviour Screening Questionnaire at ages 2 (n=537) and 4 (n=458) years, assessing sleeping, eating, encopresis, attention seeking and dependency, relations with other children, activity, concentration, control, tempers, mood, worries, and fears. A cutoff ≥10 was used to categorize high risk of behavioral problems. | Child behavior scores at age 4 were higher among children in the high (5.4) compared to low (4.4) prenatal stress group. In multivariate logistic regression analyses, prenatal stress predicted behavioral problems at age 4 (OR=2.66, CI=1.28, 5.54). Results persisted when controlling for postpartum depression.  Analyses of stressor type indicated relationships between family and marital stress and child behavior, but not economic or societal stress. | Previous studies in the U.S. and Europe had shown relationships between prenatal stress and child behavior, but this is the first to do so in Africa. This provides confirmation of findings in a setting characterized by high levels of political and societal violence. Family and marital stressors were the most important predictors of child behavior. The study did not assess stressors from birth to age 4, which might account for some of the association seen. |
| Rondó et al. 2003 (27) | Brazil (Latin America & the Caribbean, upper-middle income): Jundiaı´ (Southeast  Brazil) | 865 pregnant women (<16 wks) who attended  antenatal care from Sept 1997–Aug 2000 in 12 health units and 5 hospitals in Jundiaı´. Participants were insured by the National Health Service that assists low income families. Women with chronic infectious or metabolic diseases, cardiopathy, mental diseases, hyper-tension, pre-eclampsia, vaginal bleeding, and multiple deliveries were excluded. | Women were interviewed by psychologists at <16 pregnancy, 20-26 wks, and 30-36 wks pregnancy.  Measures included:  - Perceived  Stress Scale (PSS)  - General Health Questionnaire (GHQ), categorized as low (scores 0-3) and high (>3)  - State Trait Anxiety Inventory (STAI); cutoffs ≥40 were used to categorize state and trait anxiety | Interviewers assessed women’s medical and obstetric history (parity, prior history of LBW), maternal demographics, smoking, alcohol, and coffee intake, and pre-pregnancy weight and height. Data were verified against medical and antenatal care cards. | LBW, PTB, and intrauterine growth restriction (IUGR) were recorded from hospital records.  GA was assessed by ultrasound before 20 wks pregnancy, the Capurro method, and LMP. | Prevalence of maternal distress based on GHQ and STAI scores varied from 25.9-52.9% across pregnancy.  In multivariate logistic regression analyses, GHQ >3 in the 2^nd^ interview predicted LBW (RR=1.97, CI=1.12, 3.47), and GHQ >3 in the 3^rd^ interview predicted PTB (RR=2.32, CI=1.18, 4.60). Maternal psychosocial health did not predict IUGR. STAI and PSS scores did not predict any outcomes. | The study highlights that maternal psychosocial health, assessed by GHQ, is associated with LBW and PTB, mirroring results from other studies. However, stress and anxiety scores were not predictive. Results might suggest that the GHQ is a particularly useful measure in this setting. |
| Rondó et al. 2013 (71) | Brazil (Latin America & the Caribbean, upper-middle income): Jundiaı´ (Southeast  Brazil) | 409 women from an original sample of 865 (described above[30]) who participated assessments of child nutritional status from 2004-2006, when children were 5-8 years old. | Women were interviewed by psychologists at <16 pregnancy, 20-26 wks, and 30-36 wks, and child age 5-8 years.  Measures included those described above[30]  - Perceived  Stress Scale (PSS)  - General Health Questionnaire (GHQ)  - State Trait Anxiety Inventory (STAI) | At child age 5-8 years, interviewers assessed maternal demographics and breastfeeding duration. Maternal height and weight were measured.  During pregnancy, interviewers assessed women’s medical and obstetric history, demographics, smoking, alcohol, and coffee intake, and anthropometry; see above[30] | Child height and weight at age 5-8 years were measured, and BMI Z-scores were computed based on WHO child growth references. Children were classified as underweight (BMIZ<-2), normal weight (BMIZ=-2-1), overweight (BMIZ=1-2), or obese (BMIZ>2). | In multivariate linear regression analyses, PSS scores 5-8 years postpartum (β=-0.04) and 2^nd^T GHQ scores (β=-0.09) predicted children’s BMIZ scores. Thus, greater maternal stress and distress both during pregnancy and postpartum predicted lower BMIZ scores. | Results demonstrate that both prenatal and postpartum maternal psychosocial health is associated with child growth. The authors suggest that maternal capacity to care for children is impaired when women are not “physically or mentally well”. The authors highlight the importance of further studies of maternal distress in areas with high prevalence of child malnutrition and/or obesity. |
| Rosa et al. 2016 (72) | Mexico (Latin America & the Caribbean, upper-middle income): Mexico City | 417 women in The Programming Research in Obesity, Growth, Environment and Social Stressors (PROGRESS) study. PROGRESS recruited pregnant women (<20 wks) receiving prenatal care through the Mexican Social Security System from Jul 2007–Feb 2011. Eligible women had access to a telephone, no history of heart or kidney disease, and did not consume alcohol daily or use steroid or antiepilepsy medication. | Psychologists administered the Crisis in Family Systems Revised (CRISYS) survey during the 2^nd^ or 3^rd^T and at the 48-month visit. CRISYS assesses life events in the past 6 months from 11 domains.  Domains with 1 or more negative life events (NLEs) were summed  into an NLE domain score. | Child’s sex, mother’s age at delivery, and mother’s report of ever having asthma were collected with questionnaires. Exposure to tobacco smoke was evaluated through report of any smoker in the home. Exposure to particulate matter (PM) was estimated  using satellite-derived aerosol optical depth measurements to yield estimates of daily residential PM levels. | The Spanish version of the Int. Study of  Asthma and Allergies in Childhood questionnaire was administered at the 48-month visit. Women were asked “Has your child ever had wheezing or whistling in the chest at any time in the past?” and “…in the past 12 months?” to indicate “ever wheeze” and “current wheeze” | Controlling for confounders, prenatal NLEs predicted risk of ever wheeze (RR=1.08, CI=1.00, 1.16) and wheeze in the past 12 months (RR=1.12, CI=1.00, 1.26). Postnatal NLEs predicted risk of ever wheeze (RR=1.12, CI=1.04, 1.21) and wheeze in the past 12 months (RR=1.21, CI=1.08, 1.35). In sex-stratified analyses, this relationship was significant among only girls. | This prospective study highlights that psychosocial stress might represent a programming factor for childhood wheezing and respiratory illness. Results are strengthened by careful consideration of confounders. Results suggest that girls might be more susceptible to postnatal stress, whereas boys were more vulnerable prenatally. |
| Ross et al. 2011 (63) | Ethiopia  (Sub-Saharan Africa, low income) : Butajira Rural Health Programme | 954 mothers and singleton infants from the Perinatal Maternal Mental Disorder in Ethiopia population-based cohort study.  Eligible women were age 15-49, spoke Amharic, lived in the DSS and were in their 3^rd^T between Jul 2005 Feb 2006. See also [48] | Self-Reporting Questionnaire (SRQ-20) of common mental disorder (CMD) symptoms. A cutoff of ≥6 was used to define high levels, during pregnancy only, postnatally only, or both (”persistent”). Local women were trained to conduct interviews, which occurred at recruitment, ~2 days after birth, and 2 months after birth. | - Interviewers assessed maternal education, SES (hunger, wealth, indebtedness), environmental conditions (e.g. water supply, sanitation), use of alcohol and khat, maternal health (diarrhea, fever, and malaria), and social support  - Height and weight were measured  - Key health behaviors, including frequency of soap use, non-exclusive breastfeeding, and infants’ vaccination status were assessed | Infant illness episodes since birth, reported by the mother at the 2 mo. assessment, including diarrhea (≥3 loose or fluid stools in a 24 h period), acute respiratory illnesses (ARI: cough with associated difficulty breathing), and fever | Prevalence of high CMD symptoms was 13.8%. In multivariate regressions, persistent CMD symptoms predicted infant diarrhoea (RR=2.15, CI=1.39, 3.34). Univariate analyses showed relationships between persistent CMD and ARI (crude RR=2.24, CI=1.52, 3.30) and fever (crude RR=1.61, CI=1.10, 2.35), but results did not persist when controlling for SES and maternal ill health. | Previous studies indicate relationships between CMD and decreased health promoting behaviors such as breastfeeding and vaccination. The current study indicates effects of CMD independent of health behaviors. Authors suggest that rather than representing a confounder, maternal ill-health might mediate the relationship between CMD and infant illness: CMD symptoms might increase mother's risk of illness and thereby increase infants’ risk. |
| Rothberg et al. 1991 (47) | South Africa  (Sub-Saharan Africa, upper-middle income): Johannesburg and Baragwanath | 1197 women who delivered at Johannesburg (n=535) or Baragwanath Hospital (n=662), interviewed by social workers within 36 hours of delivery between Mar 1987-Mar 1988. Women with a history of medical or obstetric conditions associated with LBW were excluded. | Social Readjustment Rating Scale (SRRS) of life events in the year before delivery, including death of spouse, separation, jail term, illness, marriage, dismissal from work/having to leave school, and marital conflict. Women reported social support and their perception of the stress (from “no affect” to “one of the worst years of my life”) | Interviewers collected data on demographic (age, marital status), obstetric data (gravidity, history of PTB or stillbirth), and smoking; collection methods are not described. | BW; measurement methods are not detailed | Among the Johannesburg  group, controlling for covariates, greater SRRS predicted BW. The most important stressors were death of a spouse and marital separation.  SRRS scores did not predict BW in the Baragwanath group, but dismissal from work or having to leave school predicted BW. Results were more pronounced among women <age 20.  Maternal perception of stress and psycho-social support did not predict BW. | Statistical results are not described in detail, which limits evaluations and conclusions. The authors note that the lack of relationship between SRRS and BW in Baragwanath might indicate that the SRRS is not valid in the community; different priorities might result in a need to assign different scores to the stress factors. |
| Ruwanpathirana and Fernando 2014 (50) | Sri Lanka (South Asia, lower-middle income): Colombo district | 835 pregnant women and their infants - 167 SGA (cases) and 668 controls - from a sample of 1200 pregnant women recruited within the first 8 wks of amenorrhea from Jan-Jun 2010. Women not delivering at one of the 5 selected government hospitals in the area, having mental disabilities, and living in institutions  (e.g. prisons) were excluded. | Level of psychological stress assessed with the GHQ-30 (translated version), self-administered in the 2^nd^T. Women with scores >6 were categorized as having “high stress”. Tables include “T1>6” and “T2>6” but only measurements during T2 are described. | Nurses recorded socio-demographic data, obstetric and medical history, findings of clinical examinations,  urine full report and hemoglobin level, Wealth Index, home risk factors, and family history of disability and LBW. Mothers completed the Pregnancy Physical Activity Questionnaire to assess workload during pregnancy. | SGA  Data were collected via questionnaire at the first post-partum visit. The authors note that “Available records were the main sources of  information.” | High 2^nd^T stress levels predicted SGA in univariate (OR=2.17, CI=1.43, 3.30) and multi-variate (OR=1.92, CI=1.17, 3.14) analyses. Other risk factors included having no other liveborn children, inadequate pregnancy weight gain, maximum and minimum physical work, pre-pregnancy weight <38kg, pregnancy-induced hypertension, and no support from husband. | The stress analyses are not entirely clear; more detailed descriptions of the 1^st^T assessments and subsequent definition of the “low stress” category would help to clarify the results. |
| Sanchez et al. 2013 (39) | Peru  (Latin America & the Caribbean, upper-middle income): Lima | 959 women: 479 mothers of preterm (cases) and 480 mothers of term live births at the Hospital Nacional Dos de Mayo, the Instituto Nacional Materno Perinatal de Lima, and the Hospital Edgardo Rebagliati Martins in Lima, Peru, from Jan 2009-Jul 2010. | - Patient Health Questionaire-9 (PHQ-9) of depression and anxiety. Symptoms were categorized as minimal (score=0–4), mild (5–9), and moderate-severe (≥10).  - Depression Anxiety Stress Scales (DASS-21). Moderate-severe cutoffs were ≥14 for depression, ≥10 for anxiety, and ≥19 for stress. | Trained personnel used a structured questionnaire to assess maternal demographics, reproductive and medical histories, pre-pregnancy weight, prenatal care, and vitamin use, cigarette smoking, and alcohol consumption during pregnancy. Medical records were used to validate medical and reproductive history. | PTB  GA was based on LMP and confirmed by ultrasound before 20 wks | In separate logistic regression analyses controlling for confounders, predictors of PTB included PHQ-9 Depression scores (OR for moderate-severe group=3.67, CI=2.09, 6.46), and DASS-21 Depression (OR=2.90, CI=1.66, 5.04), Anxiety (OR=2.76, CI=1.83, 4.16), and Stress (OR=11.07, CI=5.64, 21.71) scores. | The study replicates among Peruvian women the results of studies in other settings showing that depressive, anxiety, and stress symptoms are associated with risk of PTB. The authors emphasize that longitudinal studies, with prospective assessment of women’s psychosocial health in ethnically and racially diverse populations, are necessary. |
| Sanguanklin et al. 2014 (52) | Thailand (East Asia & Pacific, upper-middle income): Pathum Thani province | 175 pregnant women who were between 26-38 wks of gestation during a flood. Women were part of a larger study examining job strain and psychological distress. Eligible participants were age≥18, employed full-time, had uncomplicated singleton pregnancies and no history of mental illness. Data were collected from Feb–Apr 2012, in reference to flooding from Jul 2011–Jan 2012. | - Thai version of the Center for  Epidemiological Studies Depression Scale (CES-  D). Scores ≥19 were used to categorize antenatal depression. | Questionnaires assessed sociodemographic data and pregnancy characteristics (gestational age, pregnancy intent, parity). Participants were asked whether they were displaced or lost their jobs during the flood.  The Medical Outcome Study-Social Support Survey (MOS-SSS) was used to assess availability and adequacy of social support. | BW, collected from hospital records, and LBW and PTB.  GA was calculated based on LMP or ultrasonography, recorded from hospital records. Basic analyses of relationships between depression and GA were presented, but BW was the primary outcome of interest. | 34.9% of women had CES-D ≥19.  In linear regressions, parity, job characteristics, and displacement predicted BW. Depression and social support had no main effects. Displacement and social support interacted to explain 2.3% of variance. Among displaced women, greater social support was associated with greater BW, whereas social support had no effect on BW among non-displaced women | The authors note that the prevalence of depression symptoms did not differ between displaced and nondisplaced women. This could reflect, in part, that even women who were not displaced might worry over their friends and family, and experience distress. The study cannot disentangle effects of depression from other factors that influence health during disasters, such as inadequate dietary intake and prenatal care among displaced women. |
| Santos et al. 2014 (68) | Brazil  (Latin America & the Caribbean, upper-middle income): Pelotas (urbanized city) | 4231 births in the 2004 Pelotas Birth Cohort, which enrolled all mothers of live births in the Pelotas urban area from 1 Jan–31 Dec 2004. Mothers were interviewed soon after delivery, with follow-ups at their homes at child ages 3, 12, and 50 months, and at a research clinic at 6.8 years, on average | Maternal mood symptoms during pregnancy were defined as an affirmative answer to the question “During pregnancy, did you feel depressed or have any nervous condition*?*”, asked during the perinatal interview | Interviews were used to assess sociodemographic characteristics, parity, smoking, type of delivery, breastfeeding, and hospital admission during the child’s first year of life.  BW and GA were recorded by hospital staff. Mood symptoms 3 mos. postpartum were assessed with the Self-Reporting Questionnaire (SRQ). | Psychiatric disorders in 6-year-old children was assessed through maternal report using the Development and Well-Being Assessment (DAWBA) questionnaire | 24.6% of mothers had mood symptoms during pregnancy, and 42.0% at 3 mos. postpartum. Controlling for confounders, mood symptoms during pregnancy predicted psychiatric disorders among children (OR=1.97, CI=1.60, 2.41). Results were similar for mood symptoms at 3 months postpartum (OR=2.29 CI=1.86, 2.81). | Results highlight a relationship between mood symptoms in pregnancy and child psychiatric disorders.  Maternal mood was assessed using a single question, which limits the conclusions that can be drawn. However, the authors note that this indicator was used in longitudinal studies of maternal depression and that women with chronic depression reported the highest rates of positive answers. |
| Sasaluxnanon and Kaewpornsawan 2005 (66) | Thailand (East Asia and Pacific, upper-middle income) | 241 children age 6-12 including 122 with ADHD (cases) and 119 controls with no behavioral problems. Cases were identified through the child and adolescent psychiatric unit of the Siriraj Hospital. Controls were identified from the Kositsamosorn Primary School. Children with other Axis 1 diagnoses in DSM IV TR criteria, mental retardation, or parental problems in communication (such as psychiatric disorders or deafness) were excluded. | Mothers completed a questionnaire that assessed characteristics of the pregnancy, including “emotional distress,” reported as “yes” or “no”. | Questionnaire assessments of maternal demographics and pregnancy characteristics, including medical complications, medications, smoking and alcohol use, physical injury, and labor complications; and data about the child including BW, neonatal complications, history of seizures, head trauma, encephalitis, medication and family history of ADHD. | Attention Deficit Hyperactivity Disorder (ADHD) | Emotional distress predicted ADHD in univariate (OR=4.49, CI=2.37, 8.45) and multivariate analyses (OR=2.99, CI=1.43, 5.40). Other risk factors included pregnancy complications, neonatal complications, and family history. LBW was a risk factor for ADHD in univariate analyses. | Emotional distress remained a significant predictor of ADHD in multivariate analyses including a number of other important risk factors. The study is limited by the questionnaire items (yes or no responses), and the retrospective nature of the emotional distress assessment, which might bias results. |
| Shaikh et al. 2011 (30) | Pakistan  (South Asia, lower-middle income): Kharadar and Karimabad Karachi | 125 pregnant women ages 18-40, from 28-30 wks pregnancy, who attended antenatal clinic at the Aga Khan Hospital for Women and Children in Kharadar and Karimabad Karachi from Apr–May 2010. Women with self-reported diabetes, thyroid disorder, renal or heart disease, uterine abnormality, or antidepressant use during pregnancy were excluded. | At 28-30 wks pregnancy, women completed the A-Z stress scale and the Center for Epidemiological Studies Depression (CESD) scale, and provided a blood sample for cortisol analysis. A-Z scores in the upper and lower 25^th^ percentiles were classified as high and low stress. CES-D scores ≥16 were used to categorize depression. | Maternal age, ethnicity, education, income, behavioral characteristics (nature of work, hours of walking and standing), pregnancy characteristics  (parity, number of children, sex of children at home,  history of PTB or abortion, visit to antenatal clinic, hemoglobin level). | PTB  Pregnancy outcome and delivery information was obtained from the medical records at both centers. | 19·7% of women had high stress and 40·9% had antenatal depression. There was no difference in cortisol levels, stress, or depression among mothers with PTB and those with term infants. Univariate logistic regression analyses showed no relationships between PTB and cortisol (OR per 10-point increase in cortisol=0.78, p = 0.507), high stress (OR=0.60, p=0.519) or depression (OR=1.4, p=0.697) | Questionnaires measured symptoms at a single time point and did not differentiate between acute and chronic stress, which may have influenced findings. The small number of preterm births (15 / 125) limited the possible statistical analyses.  The authors note that prevalence of stress and depression was high, suggesting the need to address maternal mental health in the population. |
| Stewart et al. 2015 (26) | Malawi  (Sub-Saharan Africa, low income): Mangochi District, southern Malawi | 1391 pregnant women (<20 wks) ages 15-49 enrolled in the iLiNS trial (International Lipid-based Nutrient Supplement study), which enrolled pregnant women attending antenatal care into one of 3 supplement groups: 1) Lipid-based nutrient supplements (LNS); 2) multiple micronutrients (MMN); or 3) iron-folic acid tablets (IFA). Women with chronic illness or pregnancy complications were excluded. | - 10-item Perceived Stress Scale (PSS) of stress over the previous month, completed at enrollment, and at 28 and 36 wks pregnancy  - Salivary cortisol at enrollment and at 28 and 36 wks pregnancy | - Maternal anthropometrics, malaria status, haemoglobin concentration, and HIV status  - Maternal sociodemographics. SES was estimated based on ownership of radio, television, cell phone, bed, mattress, bednet, and bicycle; lighting source; drinking water supply; sanitation facilities; and flooring materials.  - The Household Food Insecurity Access Scale. | Research assistants measured BW, BL, HC, and arm circumference soon after birth. WAZ, LAZ, and HCZ were computed, but the standards used are not described.  GA was confirmed by ultrasound at enrollment | Cortisol values increased during pregnancy, but PSS scores did not change. There was no correlation between PSS and cortisol.  Greater cortisol at enrolment and 36 wks predicted shorter GA. Greater cortisol at 28 wk and 36 wk predicted lower BW, with the strongest effects among primiparous women. Cortisol was not associated with WAZ, LAZ, or HCZ at any time. PSS scores at 28 wk and 36 wk predicted shorter LAZ. PSS did not predict other outcomes. | Results show that cortisol and, less consistently, perceived stress during pregnancy predicts adverse birth outcomes. The study showed no effects of nutrient supplementation on cortisol, perceived stress, or birth outcomes. Notably, only 5% of women were underweight. Thus, nutrient deficiencies may not have been severe enough to trigger elevated maternal cortisol. Elevated cortisol concentrations in the sample might reflect non-nutritional physical or psychosocial stressors. |
| Tran et al. 2014 (60) | Vietnam (East Asia and Pacific, lower-middle income): Rural northern area | 378 pregnant women recruited before 20 wks pregnancy in a prospective cohort study, and their infants. All pregnant women from 12-20 wks pregnancy and living in the selected communes during the enrolment period (Dec 2009-Jan 2010) were eligible. | Common Mental Disorders (CMDs) assessed using the EPDS-V (Edinburgh Postnatal Depression Scale - Vietnam), administered at 20 (Wave 1) and 28 wks (W2) of pregnancy, and 6 wks (W3) and 6 months (W4) postpartum. | - Self-efficacy and parenting practices, assessed at W4 using the Longitudinal Study of Australian Children Questionnaire  - Breastfeeding, assessed using a structured questionnaire  - GA and WAZ (WHO values)  - Sociodemographic data, social support, medical history, and history of violence collected at all 4 assessments  - Maternal serum ferritin and haemoglobin at W3 and W4 | Infant social-emotional development was assessed at age 6 months using the Bayley Scales of Infant and Toddler Development Social-Emotional Questionnaire | In multivariable regression models, antenatal CMDs predicted Bayley social-emotional scores (β=-3.30, CI=-6.32, -2.89). Path analyses revealed no direct effects, but a significant indirect effect (estimate=-0.61, CI=-1.17, -0.04): antenatal CMDs predicted postnatal CMDs, which then predicted social-emotional scores. Path analyses further indicated that postnatal CMDs were related to scores through self-efficacy and parenting practices. | This rigorous prospective longitudinal study highlights a role of CMDs during pregnancy on infant social-emotional development in an understudied area, and the possible pathways underlying these relationships. Authors acknowledge that cultural factors might affect questionnaire responses. They highlight the importance of CMD screening in efforts to promote early child development, and interventions focused on parenting practices. |
| Valladares et al. 2009 (28) | Nicaragu  (Latin America & the Caribbean, lower-middle income): León (Nicragua’s 2^nd^ largest city) | 147 pregnant  women (18-39 wks) age 14-40, who were recruited from a larger sample of 478 pregnant women who participated in a community  demographic surveillance in León from Nov 2002–Jan 2004 | Cortisol was measured from saliva samples collected by trained field workers at 7:00-8:00 and 14:00-15:00.  Interviewers used a questionnaire to assess intimate partner violence, emotional distress, and social resources. The authors refer to WHO multi-country studies in their questionnaire description, but details of the questionnaire are not discussed. | Maternal age, parity, SES, and urban or rural residence are discussed in Results, but their measurement is not detailed | LBW, PTB, SGA  GA was based on LMP and the Capurro method | Low social resources and high perceived stress predicted increased cortisol. Increased cortisol predicted LBW and SGA, but not PTB. Women who reported violence had greater prevalence of LBW and SGA, but not PTB. Path analyses suggested that violence predicted LBW through: 1) increased cortisol; 2) increased cortisol and subsequent lower GA; and 3) direct abdominal trauma, and subsequent lower GA. | The authors highlight maternal stress as a mechanism through which violence during pregnancy decreases BW. Describing and controlling for covariates would strengthen these conclusions. Questionnaire methods are not described in detail, which makes it difficult to compare findings with other studies. However, the authors note the strength of the interview procedure for collection of sensitive information on violence, which might not be detected with other methods. |
| Wado et al. 2014 (51) | Ethiopia (Sub-Saharan Africa, low income): rural area in the southwest | 537 mother-infant dyads in a prospective cohort study of pregnancy. Pregnant women in their 2^nd^ or 3^rd^T living in 11 villages in the Health and Demographic  Surveillance System from Jun 2012-Feb 2013 were followed  from pregnancy through delivery. Baseline surveys were conducted with 622 women. BW was measured for 537 infants. | Depressive symptoms were assessed at baseline using the Edinburgh Postnatal Depression Scale (EPDS). A cutoff of ≥13 was used to categorize depression. | - Data on pregnancy intention, happiness with pregnancy, and  husband’s perceived pregnancy intention were collected using interviewer-administered questionnaires  - Social support was assessed with the Maternity Social Support Scale  - Mid-upper arm circumference (MUAC) at baseline  - Wealth index was computed from household assets | LBW  BW was measured within 72 h of delivery by data collectors in villages | Incidence of LBW was 17.9% and was higher among women with depressive symptoms (26.2%) compared to non-symptomatic women (15.8%) (p=0.01). In multivariate regressions, antenatal depression predicted LBW (OR=1.87, CI=1.09, 3.21), controlling for social support, MUAC, and other covariates. Unwanted pregnancy also predicted LBW (OR=2.12, CI=1.05, 4.28). | Results highlight relationships between antenatal depression and LBW even controlling for important covariates. Social support represents a potential mediator. Unwanted pregnancy also contributes to risk of LBW, possibly through maternal psychosocial health, risky behaviors, and reduced social support.  Authors note that nearly nine in ten babies in Ethiopia are delivered at home and not weighted at birth, highlighting the challenges in data collection. |
| Zhang et al. 2012 (32) | China  (East Asia and Pacific, upper-middle income): Beijing | 2782 pregnant women: 1391 women who delivered preterm (cases) and 1391 age-matched controls who delivered at term immediately after a case at one of five Maternal and Child Health hospitals in Beijing from Aug 2009–Aug 2010 | Questionnaires were administered during interviews to assess stressful life events. Details of the questionnaire are not provided, but results mention hospitalization, surgery or death of family members, and family conflict | - Interviews were used to assess sociodemographic characteristics and self-evaluation of nutrition. Medical records were reviewed for pre-pregnancy height and weight, inter-pregnancy interval, gestational diabetes, and hypertension | PTB  GA was based on LMP or ultrasound before 20 wks | Stressors included hospitalization, surgery or death of family members, and family conflict. In multivariate regression analyses, stressful life events predicted PTB (OR=5.54, CI=2.34, 13.23). | Lack of details on questionnaire methods makes it difficult to compare results to other studies. Possible confounding might be suspected for hospitalization and stress, if the reason for hospitalization is associated with increased PTB risk. The factors identified might guide more detailed studies in the future. |
| Zhu et al. 2014 (61) | China  (East Asia and Pacific, upper-middle income): Hefei | 152 pregnant women: 38 exposed to severe life events in the 1^st^T (cases) and 114 unexposed controls matched for age, education, income, delivery mode, and infant sex, identified from studies of 2552 women who had 3^rd^T prenatal visit at Hefei Hospital from Mar-Nov 2008 (see also [33]). Exclusion criteria included history of atypical pregnancy outcomes, age ≥35, cognitive disorder, pregnancy complications, multiple gestation, assisted reproductive technology, and PTB, LBW, Apgar <7, SGA, or birth defects | 19-item Prenatal  Life Events  Checklist, based on the Life Events Scale, completed at 32 wks pregnancy. Women indicated whether each event occurred during the 1^st^, 2^nd^, or 3^rd^T. | Interviews were used to assess  sociodemographic characteristics, pregnancy history, and alcohol and smoking.  Information about pregnancy complications and birth outcomes were obtained from medical charts.  Data on breastfeeding were collected during interviews at 6, 16, and 18 months postpartum  Postnatal depressive symptoms were assessed at 2 mos. postpartum using the Chinese version of the Edinburgh Postnatal  Depression Scale. | At 16 and 18 months, infant cognitive development was assessed using the Bayley Scales of Infant Dev., China Revision, including the mental dev. index (MDI) and psychomotor dev. index (PDI)  At 16 and 18 months, infant temperament was assessed using the Toddler Temperament  Scale (TTS) | Common stressful life events included living apart from partner (31.6% of cases), poor marital relationship (26.3%), housing stress (21.1%), moving to a new home (15.8%), and arguments with partner (10.5%). Controlling for confounders, exposure to life events in the 1^st^T predicted lower MDI scores (adjusted mean=103.11 among cases, 110.09 among controls). Life events did not predict PDI scores. 1^st^T life events predicted higher TTS regularity (adjusted means=2.77 vs. 2.52) and persistence and attention span scores (adjusted means=3.61 vs. 3.35). | Results suggest lower mental development, and higher scores for regularity and persistence and attention span, indicating a less optimal behavioral response among exposed infants. The strict selection criteria increase the strength of conclusions that these effects are not the result of pregnancy complications or adverse birth outcomes. The authors thus recommend interventions to help pregnant women manage psychological stress as an integral part of antenatal and postnatal care, even among women with healthy pregnancies. |
| Zhu et al. 2010 (33) | China  (East Asia and Pacific, upper-middle income): Hefei | 1800 pregnant women (3^rd^T) recruited from 3316 married pregnant women who had 3^rd^T prenatal visit at Hefei Hospital from Mar-Nov 2008 (see also [61]). Women ≥35 years, who delivered before 32 wks or had medically indicated PTB, stillbirth, birth defect, mental disorders, pregnancy complications, or assisted reproductive technology were excluded. | 19-item inventory of stressful life events.  Participants were asked to indicate whether each even had occurred in the 1^st^, 2^nd^, or 3^rd^T of pregnancy and, if so, to rate its impact, from no impact (0) to extreme impact (4). The sum of the event ratings was used to assess perception of stress. | - SES (income,  education)  - Social support based on The Chinese Revised Edition of the Social Support Scale, categorized as lower support, medium-low support, medium- high support, and high support by using quartiles.  - Coping Style Questionnaire (Chinese Revised Edition) | PTB, BW  Gestational age was based on LMP | Life events predicted PTB in the 1^st^T (RR=2.60, CI=1.29, 5.22) and 2^nd^T (RR=2.86, CI=1.32, 6.22), but not the 3^rd^T. Relationships persisted when controlling for confounders. Life events during the 1^st^T but not the 2^nd^ and 3^rd^, predicted BW (RR=–122.97, CI=–166.64, -79.29). Results persisted when adjusting for covariates. | Although others have noted effects of social support and coping styles on birth outcomes, these were not associated with PTB or BW in the current study. The authors suggest that the buffering effects of social support and coping might be limited among women experiencing severe stressors. Furthermore, cultural differences that affect coping and social support complicate comparisons among countries. |

Abbreviations

1^st^T, 2^nd^T, 3^rd^T: first, second, and third trimester

ADHD: Attention Deficit Hyperactivity Disorder

BDI: Beck Depression Inventory

BL: Birth length

BMI: Body Mass Index (kg/m^2^)

BMIZ: Body mass index Z-score

BP: Blood pressure

BW: Birth weight

CBCL: Child Behavior Checklist

CESD: Center for Epidemiological Studies Depression Scale

CI: Confidence interval

CMD: Common Mental Disorder

DASS: Depression Anxiety Stress Scales

DSM: Diagnostic and Statistical Manual of Mental Disorders

EPDS: Edinburgh Postnatal Depression Scale

EPO: Erythropoietin

GA: Gestational age

GAD: Generalized Anxiety Disorder Scale

GHQ: General Health Questionnaire

HC: Head circumference

HCAZ: Head circumference for age Z-score

HCZ: Head circumference Z-score

HR: Heart rate

IPV: Intimate partner violence

IUGR: Intra-uterine growth restriction

LAZ: Length for age Z-score

LBW: Low birthweight

LMIC: Low- and middle-income countries

LMP: Last menstrual period

MLEI: Modified Life Events Inventory

OR: Odds ratio

PHQ: Patient Health Questionnaire

PLBW: Preterm low birthweight

PSS: Perceived Stress Scale

PTB: Preterm birth

PTSD: Posttraumatic stress disorder

RR: Relative risk

SES: Socioeconomic status

SGA: Small for gestational age

SRQ: Self-Reporting Questionnaire

SRRS: Social Readjustment Rating Scale

STAI: State-Trait Anxiety Inventory

TTS: Toddler Temperament Scale

WAZ: Weight for age Z-score

**Additional References**

80. Thurston SW, Ryan L, Christiani DC, Snow R, Carlson J, You L, Cui S, Ma G,

Wang L, Huang Y, Xu X. Petrochemical exposure and menstrual disturbances. Am J Ind Med. 2000;38(5):555-64.

81. Christiani DC, Niu T, Xu X. Occupational Stress and Dysmenorrhea in Women Working in Cotton Textile Mills. Int J Occup Environ Health. 1995;1(1):9-15.
