# Supplementary material for: Prenatal stress and child development: A scoping review of research in low- and middle-income countries
Source: PLoS One. 2018 Dec 28;13(12):e0207235. doi: 10.1371/journal.pone.0207235 (PMC6310253; doi:10.1371/journal.pone.0207235)
Supplement: S2 Appendix — (DOCX) [file pone.0207235.s004.docx]

**Appendix S2:**  Assessment of methodological quality

Methodological quality was assessed using items from the Quality Assessment Tool for Quantitative Studies, developed by the Effective Public Health Practice Project (https://www.nccmt.ca/knowledge-repositories/search/14). Criteria assessed included selection bias, confounders, data collection methods (distinguished for independent and dependent variables), and analysis.

Presentation of descriptive statistics for covariates discussed, with comparison among groups (if applicable), was considered a strength. The use of validated stress questionnaires completed prospectively was considered a strength, whereas the use of non-validated questionnaires or retrospective data collection was considered a weakness. Collection of data on birthweight and gestational age from medical records as opposed to maternal report was considered a strength. Similarly, measurement of birthweight at birth as opposed to in the following days was considered a strength. Collection of data on covariates related to medical history (such as obstetric complications) from medical records as opposed to self-report was considered a strength. Controlled time of sample collection for biomarkers with diurnal variation (such as salivary cortisol) was a strength, whereas a lack of control for or specification of time of sample collection was considered a weakness.

|  | Selection bias | Confounders | Data collection methods | | Analysis |
| --- | --- | --- | --- | --- | --- |
| Study |  |  | Independent variable(s) | Dependent variable(s) |  |
| Abeysena et al. 2010 (35) | Moderate | Moderate | Strong | Strong | Strong |
| Abramson et al. 1961 (62) | Weak | Moderate | Moderate | Moderate | Weak |
| Arffin et al. 2012 (56) | Weak | Weak | Weak | Moderate | Weak |
| Arteaga-Guerra et al. 2010 (29) | Weak | Weak | Weak | Strong | Weak |
| Baig et al. 2013 (43) | Moderate | Moderate | Weak | Strong | Weak |
| Barrios et al. 2014 (31) | Strong | Moderate | Moderate | Moderate | Strong |
| Başgül et al. 2011 (69) | Moderate | Moderate | Weak | Strong | Weak |
| Bhat et al. 2015 (59) | Moderate | Moderate | Strong | Moderate | Moderate |
| Bindt et al. 2013 (40) | Moderate | Moderate | Strong | Strong | Moderate |
| Brittain et al. 2015 (41) | Strong | Moderate | Strong | Strong | Strong |
| Cerón-Mireles et al. 1996 (44) | Strong | Moderate | Weak | Strong | Strong |
| Chen et al. 2000 (53) | Moderate | Moderate | Weak | Strong | Strong |
| Christian et al. 2016 (36) | Moderate | Moderate | Moderate | Strong | Strong |
| Fan et al. 2016 (70) | Moderate | Weak | Strong | Strong | Moderate |
| Frith et al. 2015 (37) | Strong | Moderate | Strong | Moderate | Strong |
| Hanlon et al. 2009 (48) | Moderate | Moderate | Strong | Moderate | Moderate |
| Isaksson et al. 2015 (64) | Weak | Moderate | Strong | Strong | Moderate |
| Karamoozian & Askarizaden 2015 (54) | Moderate | Weak | Moderate | Moderate | Weak |
| Kertes et al. 2016 (57) | Weak | Weak | Strong | Strong | Strong |
| Koen et al. 2016 (45) | Moderate | Moderate | Moderate | Strong | Moderate |
| Meghea et al. 2014 (25) | Weak | Moderate | Moderate | Weak | Moderate |
| Mirabzadeh et al. 2013 (34) | Strong | Moderate | Strong | Strong | Strong |
| Mulligan et al. 2012 (58) | Weak | Weak | Strong | Strong | Strong |
| Nasiri et al. 2010 (38) | Strong | Moderate | Strong | Strong | Moderate |
| Nasreen et al. 2010 (49) | Moderate | Moderate | Strong | Moderate | Strong |
| Nepomnaschy et al. 2006 (55) | Moderate | Moderate | Strong | Strong | Strong |
| Pires et al. 2013 (65) | Moderate | Moderate | Weak | Strong | Moderate |
| Qiao et al. 2012 (42) | Strong | Moderate | Moderate | Moderate | Weak |
| Qu et al. 2016 (46) | Moderate | Moderate | Moderate | Moderate | Weak |
| Ramchandani et al. 2010 (67) | Weak | Moderate | Moderate | Strong | Strong |
| Rondó et al. 2003 (27) | Moderate | Moderate | Strong | Strong | Strong |
| Rondó et al. 2013 (71) | Moderate | Moderate | Strong | Strong | Strong |
| Rosa et al. 2016 (72) | Weak | Moderate | Strong | Moderate | Strong |
| Ross et al. 2011 (63) | Strong | Moderate | Strong | Weak | Moderate |
| Rothberg et al. 1991 (47) | Moderate | Weak | Weak | Weak | Weak |
| Ruwanpathirana & Fernando 2014 (50) | Strong | Moderate | Moderate | Weak | Moderate |
| Sanchez et al. 2013 (39) | Strong | Moderate | Weak | Strong | Strong |
| Sanguanklin et al. 2014 (52) | Moderate | Moderate | Strong | Strong | Moderate |
| Santos et al. 2014 (68) | Strong | Strong | Weak | Moderate | Moderate |
| Sasaluxnanon & Kaewpornsawan 2005 (66) | Weak | Weak | Weak | Moderate | Weak |
| Shaikh et al. 2011 (30) | Moderate | Moderate | Strong | Strong | Weak |
| Stewart et al. 2015 (26) | Strong | Moderate | Strong | Moderate | Strong |
| Tran et al. 2014 (60) | Moderate | Moderate | Strong | Moderate | Strong |
| Valladares et al. 2009 (28) | Weak | Weak | Strong | Moderate | Strong |
| Wado et al. 2014 (51) | Moderate | Moderate | Strong | Moderate | Strong |
| Zhang et al. 2012 (32) | Strong | Moderate | Weak | Strong | Moderate |
| Zhu et al. 2014 (61) | Weak | Moderate | Moderate | Strong | Moderate |
| Zhu et al. 2010 (33) | Strong | Moderate | Moderate | Strong | Moderate |
